# Analysis of the Phospholipid Profile of Metaphase II Mouse Oocytes Undergoing Vitrification

**DOI:** 10.1371/journal.pone.0102620

**Published:** 2014-07-17

**Authors:** Jaehun Jung, Hyejin Shin, Soyoung Bang, Hyuck Jun Mok, Chang Suk Suh, Kwang Pyo Kim, Hyunjung Jade Lim

**Affiliations:** 1 Department of Pharmacology, Konkuk University School of Medicine, Seoul, Korea; 2 Department of Biomedical Science & Technology, Institute of Biomedical Science & Technology, Konkuk University, Seoul, Korea; 3 Department of Applied Chemistry, College of Applied Science, Kyung Hee University, Yongin, Gyeonggi-do, Korea; 4 Department of Obstetrics and Gynecology, Seoul National University Bundang Hospital, Seongnam, Gyeonggi-do, Korea; The Ohio State University, United States of America

## Abstract

Oocyte freezing confers thermal and chemical stress upon the oolemma and various other intracellular structures due to the formation of ice crystals. The lipid profiles of oocytes and embryos are closely associated with both, the degrees of their membrane fluidity, as well as the degree of chilling and freezing injuries that may occur during cryopreservation. In spite of the importance of lipids in the process of cryopreservation, the phospholipid status in oocytes and embryos before and after freezing has not been investigated. In this study, we employed mass spectrometric analysis to examine if vitrification has an effect on the phospholipid profiles of mouse oocytes. Freshly prepared metaphase II mouse oocytes were vitrified using copper grids and stored in liquid nitrogen for 2 weeks. Fresh and vitrified-warmed oocytes were subjected to phospholipid extraction procedure. Mass spectrometric analyses revealed that multiple species of phospholipids are reduced in vitrified-warmed oocytes. LIFT analyses identified 31 underexpressed and 5 overexpressed phospholipids in vitrified mouse oocytes. The intensities of phosphatidylinositol (PI) {18∶2/16∶0} [M−H]− and phosphatidylglycerol (PG) {14∶0/18∶2} [M−H]− were decreased the most with fold changes of 30.5 and 19.1 in negative ion mode, respectively. Several sphingomyelins (SM) including SM {d38∶3} [M+H]+ and SM {d34∶0} [M+K]+ were decreased significantly in positive ion mode. Overall, the declining trend of multiple phospholipids demonstrates that vitrification has a marked effect on phospholipid profiles of oocytes. These results show that the identified phospholipids can be used as potential biomarkers of oocyte undergoing vitrification and will allow for the development of strategies to preserve phospholipids during oocyte cryopreservation.

## Introduction

The management of female fertility through the cryopreservation of oocytes or ovarian tissue has many advantages. Cryopreservation can be used to keep extra oocytes after an in vitro fertilization (IVF) cycle and to preserve the oocytes of cancer patients or women who wish to store their oocytes for social reasons [1], [2]. Based on the clinical outcomes of several studies, the most recent guidelines on mature oocyte cryopreservation recommend that oocyte vitrification and warming should no longer be considered an experimental practice based on clinical outcomes of several studies [3]. Thus, the general need for cryopreservation of oocyte or ovarian tissue is expected to continue to increase. In this context, it is necessary to fine-tune current cryopreservation techniques by using novel biochemical and molecular parameters. Two basic methods have been established for oocyte cryopreservation, slow-freezing and vitrification [1]. For human oocytes, both of these methods are used in IVF clinics world-wide [3].

Oocyte freezing causes thermal and chemical stress in the oolemma and various other intracellular structures due to the formation of ice crystals. Permeating cryoprotectants (CPs), such as dimethyl sulfoxide (DMSO), ethylene glycol (EG), and propylene glycol (PEG), are generally used in combination with non-permeating sugar-type CPs in the vitrification solution to minimize such stresses [4]. Lipids are fundamental macromolecules in cells, constituting the membranes of various organelles and the plasma membrane [5]. They are also directly involved in signal transduction in the form of lipid mediators including phosphatidylinositols, phosphoinositides, sphingolipids, and eicosanoids [6]. Lipids also give rise to numerous types of small vesicles within cells that are involved in protein transport, autophagy, protein degradation, and other subcellular processes [5]. Membrane lipids, composed mainly of phospholipids, glycolipids, and cholesterol, undergo a lipid phase transition (LPT) during chilling [7]. Membrane fluidity is tightly correlated with the composition of membrane lipids, a connection that is reflected in the difference in the LPT temperatures of distinct membranes [8]. There is a correlation between survival rates after cryopreservation and LPT temperatures. For example, human zygotes with low LPT temperature show a higher survival rate than oocytes with high LPT temperatures [7]. Chilling injury is known to compromise the membrane integrity of bovine oocytes [9]. Therefore, the lipid profiles of oocytes and embryos are closely associated with the degree of chilling and freezing injuries incurred during cryopreservation [7]. Despite the importance of lipids during cryopreservation, the status of phospholipids in oocytes and embryos before and after freezing has not been investigated.

Mass spectrometry is an integral technique used in lipidomics, a systemic approach to elucidate the lipid levels and composition in cells under various physiological and pathological conditions [10], [11]. Lipid composition in oocytes and preimplantation embryos has been examined in mice and other species [12]–[15]; however, there is no data on whether the composition of phospholipids within these systems is affected by the cryopreservation process. In various disciplines, the results obtained using matrix-assisted laser desorption/ionization time-of-flight mass spectrometry (MALDI-TOF MS) have produced better diagnostic efficacy than other established diagnostic tools. MALDI-TOF MS is a fast, sensitive, and highly reliable method to analyze changes in macromolecules in small quantities of biological material. In this study, we analyzed the phospholipid profile in mouse oocytes prior to and following vitrification by using MALDI-TOF MS. Our purpose in this study was to determine whether the vitrification-warming process affected the phospholipid profile in oocytes. To the best of our knowledge, we are the first to demonstrate that specific phospholipids are reduced or increased in mouse oocytes after cryopreservation and that the mass peaks of phospholipids are potential biomarkers of oocyte undergoing vitrification.

## Results

### Visualization of fatty acids and plasma membrane in mouse oocytes

**Figure 1:**
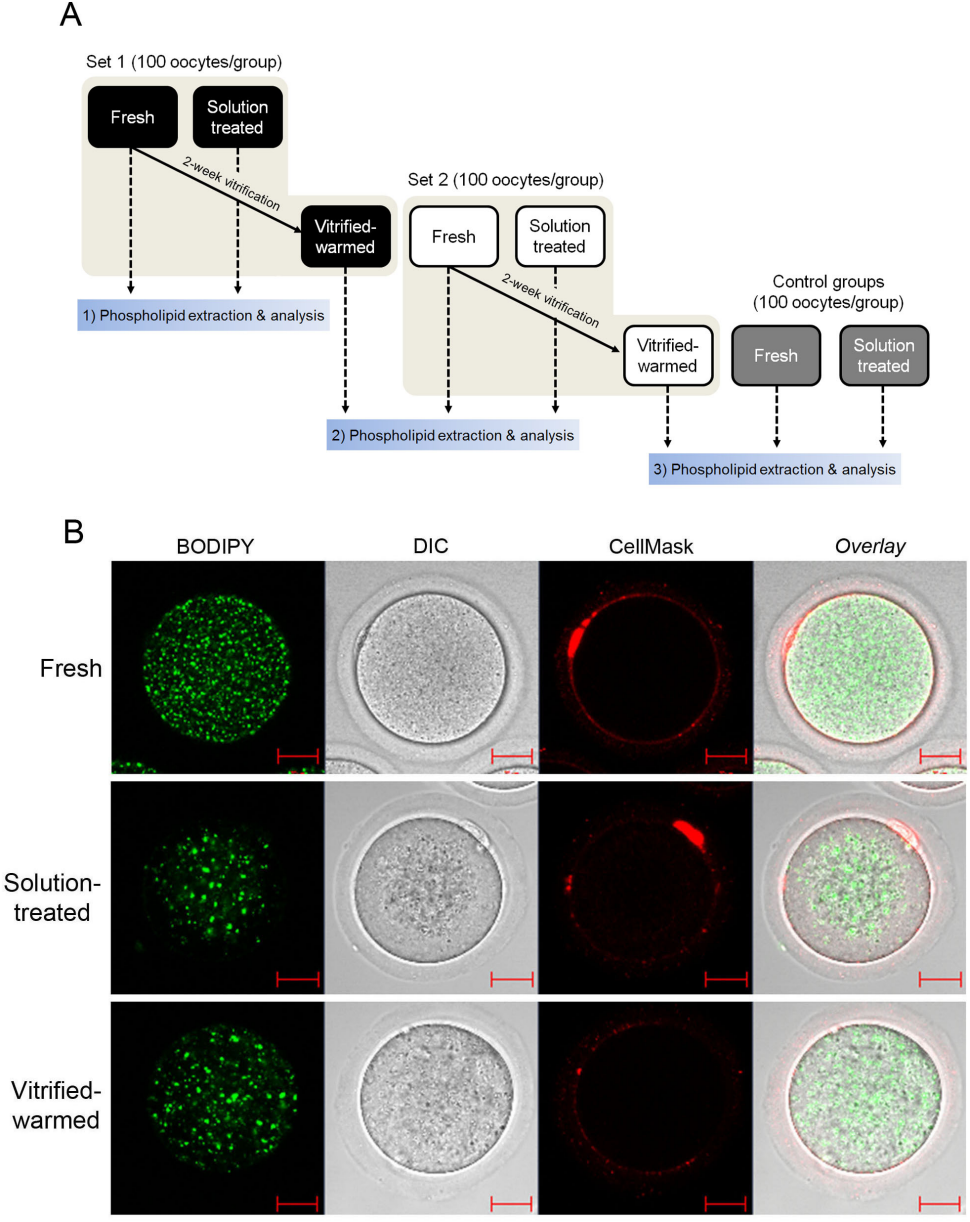
Experimental design and BODIPY/CellMask staining of mouse oocytes prior to and after vitrification. (A) A schematic diagram showing the experimental design and the three experimental groups. Two sets of vitrified-warmed oocytes with matching controls (represented as Set 1 and Set 2 in the diagram) were used. Since phospholipid extraction and subsequent analyses were performed on the day of oocyte preparation, the 2^nd^ control groups were prepared on the day of thawing vitrified oocytes of the previous set. Another set of control oocytes were prepared when extracting phospholipids from vitrified-warmed oocytes of the 2^nd^ set. This was added to ensure the quality of phospholipid extraction and analyses. Mass spectrometric analyses were performed in all groups shown. For statistical analysis, two full sets of data (shown in beige areas) excluding the last set of controls (shown in gray boxes) were included. (B) Fresh MII oocytes, solution-treated oocytes, and vitrified-warmed oocytes (2-week vitrification) were stained with BODIPY 500/510 (10 µg/ml) and CellMask (2.5 µg/ml). Stained oocytes were washed with media and examined under a confocal microscope without fixation. BODIPY and CellMask are shown in green and red, respectively. The experiment was repeated more than three times with different pools of oocytes. Representative images are shown. Red scale bar, 20 µm.

To obtain general idea if vitrification affect the status of lipid-enriched subcellular structures in mouse MII oocytes is affected by vitrification, we stained them with two vital dyes. BODIPY 500/510, a fluorescent fatty acid analog, and CellMask deep red stain labeled intracellular fatty acids and plasma membranes, respectively. As shown in Fig. 1B, fresh mouse MII oocytes possess numerous small lipid-containing vesicular structures (green) uniformly distributed throughout the ooplasm. In contrast, BODIPY-labeled structures tended to be larger and more condensed in solution-treated and vitrified-warmed oocytes. CellMask uniformly stained the periphery of fresh oocytes. In contrast, this staining pattern was less uniform in solution-treated and vitrified-warmed oocytes (Fig. 1). Taken together, these data indicate that CP-containing solutions cause an immediate osmotic shock and that lipid-containing subcellular structures and plasma membrane rapidly respond to this osmotic stress. Thus, this result provides a clue that vitrification process may alter various lipid-containing subcellular structures and phospholipid components of plasma membrane.

### Phospholipid analyses of vitrified-warmed oocytes

To this end, we sought to investigate if quantifiable changes in lipid profiles occur in mouse oocytes after vitrification. Because lipid composition affects LPT upon chilling and freezing [8], it is important to investigate vitrification-associated changes in oocyte phospholipid composition. Thus, we analyzed phospholipid profiles of mouse oocytes before and after vitrification using MALDI-MS. A group of oocytes were treated only with vitrification and warming solutions was included as control (Fig. 1A). A schematic diagram of the procedure is shown in Fig. 1A. To distinguish background peaks generated by the matrix, pure matrix profiles of both the binary and 9-aminoacridine matrices were determined (Fig. S1).

**Figure 2:**
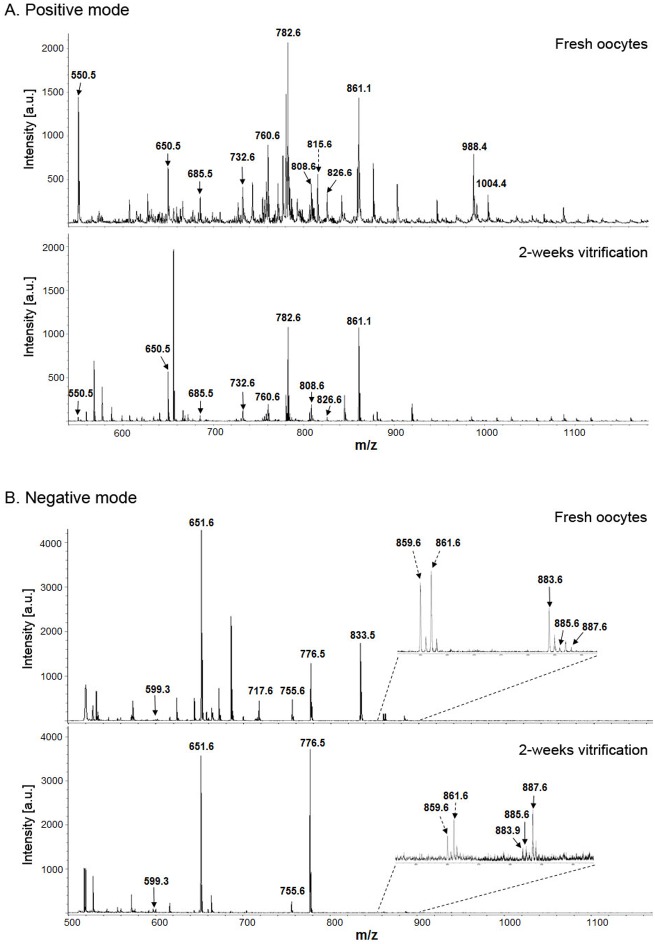
Representative mass spectra of lipids obtained from fresh and vitrified mouse oocytes in (A) positive ion mode and in (B) negative ion mode. m/z, mass-to-charge ratio; au, arbitrary unit.

We compared representative MALDI spectra obtained for the phospholipid extracts of fresh and vitrified oocytes in positive ion mode (Fig. 2A) and negative ion mode (Fig. 2B). As shown in Fig. 2A, the majority of phospholipid peaks, including major mass peaks m/z 760.6 and m/z 782.6, were reduced in the vitrification group. From two biological replicates (total 200 oocytes in each group), spectra were obtained from 20 technical replicates and 15 technical replicates in positive and negative ion modes, respectively, with reproducible results. The spectra of vitrified oocytes, showed an overall decreasing trend for the majority of mass peaks. Thus, the phospholipid profiling of oocytes with MALDI-MS is reproducible and comparable in terms of m/z values and signal intensity.

**Figure 3:**
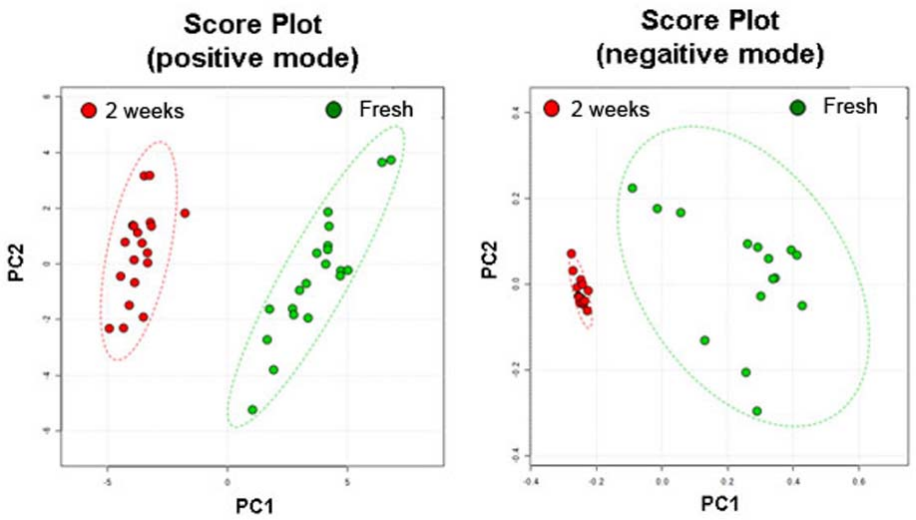
Principal component analysis plots for the phospholipid mass spectra. Fresh oocytes, green dots; 2-week vitrification, red dots.

**Figure 4:**
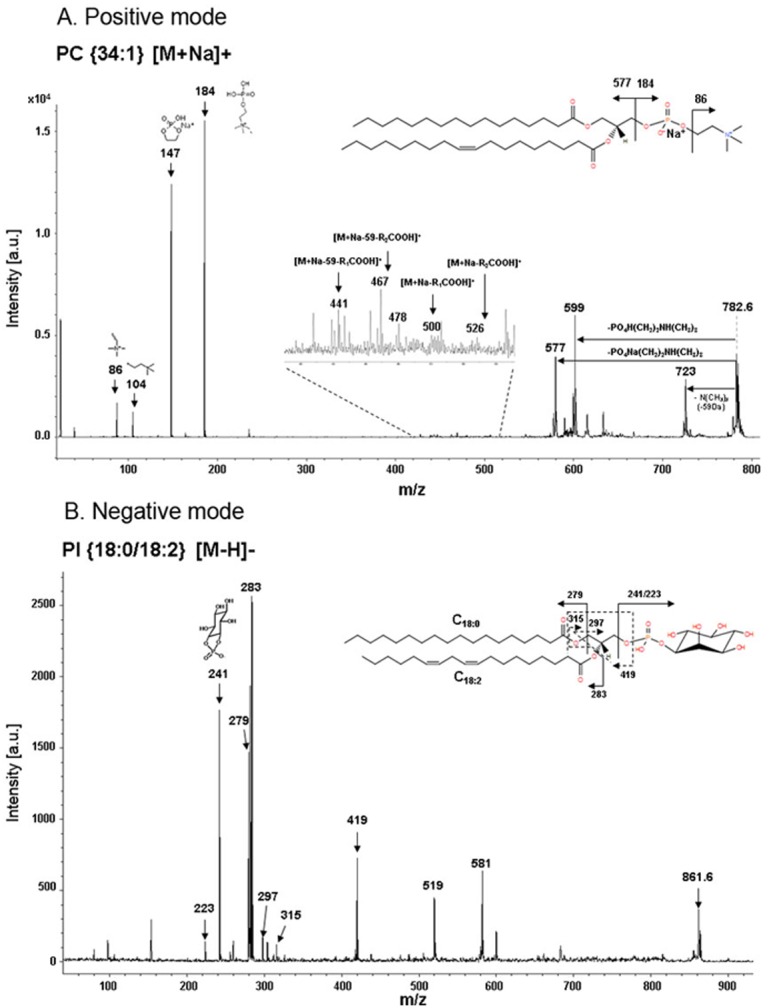
Fragmentation spectra of representative phospholipid species by using the LIFT technique. (A) m/z 782.6; PC {34∶1} [M+Na]+ in positive ion mode and (B) m/z 861.6; PI {18∶0/18∶2} [M−H]− in negative ion mode. Fragmentation spectra for other phospholipids are shown in Fig. S3.

We next performed PCA using ClinProTools 2.2 software (Fig. 3) [16]. Total MALDI-MS datasets acquired in positive and negative ionization modes were analyzed. A number of m/z values showed significant distribution changes in oocyte phospholipid extracts. As shown in Fig. 3, fresh and vitrified oocytes were separated by a large gap in PCA plots. The MALDI spectra of phospholipids can separate fresh and vitrified oocytes with a 95% confidence interval. To determine whether CP-induced osmotic shock affects the phospholipid profiles of oocytes, we examined PCA plots of solution-treated oocytes. As shown in Fig. S2, the fresh and solution-treated oocyte plots were closely situated, but were separate from that of vitrified oocytes. Representative spectra from fresh and solution-treated oocytes were also similar (Fig. S2). Thus, the vitrification process significantly changed the phospholipid profile of oocytes; however, the vitrification and warming solutions themselves did not have noticeable effect on mass spectra (Fig. S2). While the vitrification solution-associated osmotic changes may temporarily alter the organization of fatty acid-enriched subcellular structures (Fig. 1B), it does not appear to cause quantifiable fluctuations in the phospholipid pool. Fig. 4 shows representative fragmentation spectra in positive and negative ionization modes. The fragmentation spectrum of m/z 782.6; {PC 34∶1} [M+Na]+ showed peaks corresponding to specific choline head groups containing phosphate (m/z 184), sodium adducted phosphate (m/z 147), and other distinct peaks corresponding to phospholipids (Fig. 4A). Fig. 4B shows the fragmentation spectrum of m/z 861.6. The fragmentation peaks contained a phosphatidylinositol head group (m/z 241) and fatty acid chains. The other identified phospholipids were annotated using LIFT mode and their fragmentation spectra are shown in Fig. S3.

**Figure 5:**
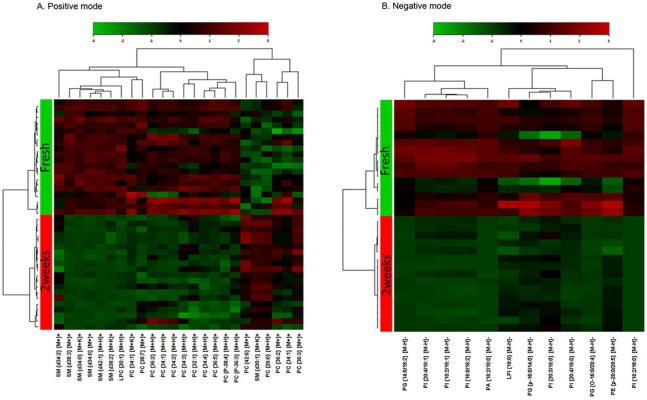
Hierarchical clustering of each sample data set showing differentially expressed phospholipids. (A) Positive ion mode. (B) Negative ion mode.

Hierarchical cluster analysis (Fig. 5) [17] of the acquired MALDI-MS spectra also clearly suggest that the average peak intensities extracted from each set are well separated and easily distinguishable between fresh and vitrified oocytes. Our data provide putative biomarker peaks for phospholipids that show vitrification-induced changes. These peaks were identified with confidence using direct in situ analysis by using MALDI LIFT MS (Table 1).

**Table 1:** Differentially expressed phospholipids between fresh oocytes and vitrified oocytes (2 weeks).

Underexpressedphospholipids invitrified oocytes				Overexpressedphospholipids invitrified oocytes			
*m/z*	*P* *	*Ratio* *	*Assignment*	*m/z*	*P*	*Ratio*	*Assignment*
*Positive* *mode*				*Positive* *mode*			
777.5	4.48E-23	10.44	SM {d38∶3} [M+Na]+	808.6	2.86E-01	0.96	PC {36∶2} [M+Na]+
743.6	1.53E-23	9.78	SM {d34∶0} [M+K]+	806.6	2.99E-04	0.86	PC {36∶3} [M+Na]+
815.6	1.12E-27	3.58	SM {d42∶1} [M+H]+	884.6	5.05E-07	0.82	PC {42∶6} [M+Na]+
550.5	4.72E-22	3.05	LPC {20∶1} [M+H]+	650.5	4.90E-10	0.69	PC {26∶0} [M+H]+
727.5	6.57E-23	2.42	SM {d34∶0} [M+Na]+	685.5	1.20E-13	0.61	SM {d30∶1} [M+K]+
795.6	1.84E-22	2.16	SM {d38∶2} [M+K]+				
798.6	4.16E-10	1.88	PC {34∶1} [M+K]+				
780.6	1.33E-14	1.66	PC {36∶5} [M+H]+				
754.6	1.29E-13	1.65	PC {34∶4} [M+H]+				
794.6	7.21E-15	1.63	PC {p-38∶4} [M+H]+				
826.6	1.95E-10	1.61	PC {38∶7} [M+Na]+				
758.6	6.28E-09	1.48	PC {34∶2} [M+H]+				
760.6	1.25E-06	1.42	PC {34∶1} [M+H]+				
786.6	2.33E-05	1.35	PC {36∶2} [M+H]+				
768.6	4.45E-07	1.25	PC {p-36∶3} [M+H]+				
756.6	3.05E-07	1.24	PC {34∶3} [M+H]+				
732.6	0.000019421	1.18	PC {32∶1} [M+H]+				
723.5	6.6907E-06	1.17	SM {d34∶2} [M+Na]+				
782.6	0.2259	1.04	PC {34∶1} [M+Na]+				
***Negative*** ***mode***							
833.6	5.93E-17	30.52	PI {18∶2/16∶0} [M−H]−				
717.6	6.68E-11	19.17	PG {14∶0/18∶2} [M−H]−				
699.5	2.54E-17	7.48	PA {18∶2/18∶0} [M−H]−				
883.6	3.82E-09	4.38	PI {20∶4/18∶1} [M−H]−				
859.6	1.32E-08	3.42	PI {18∶2/18∶1} [M−H]−				
755.6	3.39E-11	2.88	PG {O-16∶0/20∶4} [M−H]−				
861.6	6.85E-09	2.82	PI {18∶0/18∶2} [M−H]−				
885.6	3.41E-05	1.91	PI {20∶4/18∶0} [M−H]−				
677.7	5.27E-03	1.60	PG {p-16∶0/14∶0} [M−H]−				
599.3	1.59E-05	1.50	LPI {18∶0} [M−H]−				
776.6	6.22E-04	1.29	PE {p-20∶0/20∶5} [M−H]−				
887.6	1.41E-01	1.18	PI {20∶3/18∶0} [M−H]−				

*Ratio, Fresh oocytes/2 weeks vitrified oocytes.

*P, *p* value.

*PC, Phosphatidylcholines.

*SM, Sphingomyelins.

*LPC, Lysophosphatidylcholine.

*PI, Phosphatidylinositol.

*PG, Phosphatidylglycerol.

*LPI, Lysophosphatidylinositol.

*PE, Phosphatidylethanolamine.

**Figure 6:**
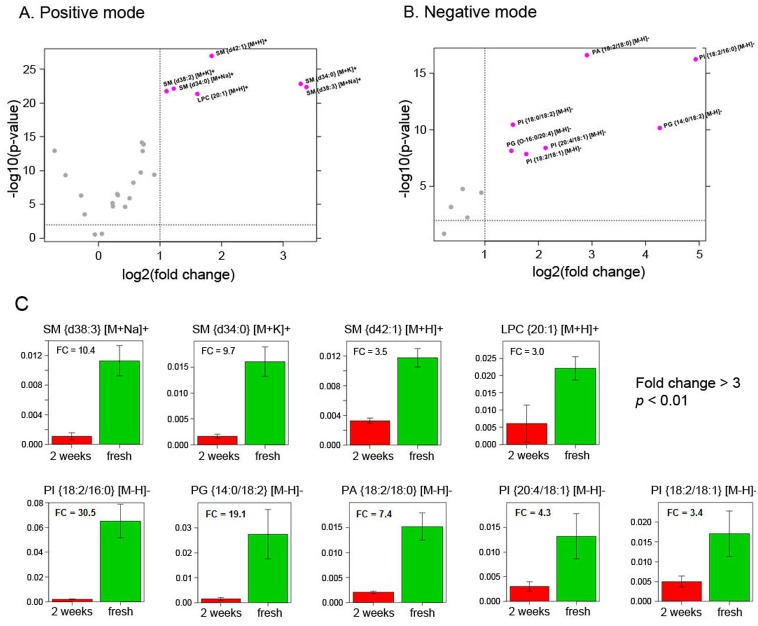
Relative intensities of differentially expressed phospholipids in fresh and vitrified oocytes measured by MALDI-TOF MS in (A) positive ion mode and in (B) negative ion mode. Red dots, phospholipid species with fold changes greater than 2 (*p*<0.01). (C) Phospholipids that are significantly reduced after vitrification and warming. Fold changes greater than 3 are shown (*p*<0.01).

The MALDI-MS analysis of lipid extracts of fresh and vitrified oocytes showed 36 major peaks common to the two datasets. In vitrified oocytes, 19 and 5 mass peaks were significantly decreased and increased in positive ion mode, respectively. In negative ion mode, 12 mass peaks had decreased (Table 1). Fig. 6C shows 9 phospholipids that were significantly reduced in vitrified oocytes (fold change >3, *p*<0.01). Phospholipids including phosphatidylcholines (PC), SM, PG, PI, and lysophosphatidylcholine (LPC) species were affected by vitrification. In positive ionization mode, SM {d38∶3} [M+Na]+ and SM {d34∶0} [M+K]+ showed the greatest reduction. In negative ionization mode, the intensities of PI {18∶2/16∶0} [M−H]− and PG {14∶0/18∶2} [M−H]− decreased the most with fold changes of 30.5 and 19.1, respectively. Overall, the downward trend of multiple phospholipids demonstrates that vitrification can have a marked effect on lipid degradation.

## Discussion

Endogenous lipids in oocytes and early preimplantation embryos are considered to be an energy substrate [18]. Lipid content varies greatly in mammalian oocytes. Mouse oocytes, for example, have a relatively lower fat content than domestic species [18], [19]. Lipids play both structural and metabolic roles in mammalian oocytes. Therefore, the level of lipids within the oocyte may reflect the degree of biochemical and physical stress it endures during cryopreservation. By using MALDI-MS, we demonstrated that phospholipids in oocytes undergo dynamic changes during cryostorage. Several major phospholipid species identified in the mass spectra (Fig. 2, Table 1) have been identified as predominant species in other mammalian oocytes [12], [13], [15], confirming that the conditions of our analyses produced valid and reliable outputs. Of these major phospholipids, 31 phospholipids decreased and 5 increased in mouse oocytes in response to vitrification. Nine phospholipids showed a significant reduction in oocytes after vitrification (Fig. 6). In the mass spectra, major peaks including m/z 859.6, 903.7, 947.7, and 991.7 were identified as triglycerides. These peaks were also underexpressed in vitrified oocytes (data not shown). Therefore, our study was focused on the characterization of phospholipids, but we also confirmed that several species of triglycerides were reduced in vitrified oocytes. Whether these specific changes in phospholipid profile are more pronounced with longer storage periods (i.e., several months or years) shall make for interesting study. Further investigation is required to determine whether the method of slow freezing or the composition of CPs differentially affect phospholipid profiles.

In positive ionization mode, several sphingomyelins show large degree of reduction in vitrified oocytes when compared to fresh oocytes (Fig. 6). Several reports suggested that sphingomyelins exhibit high affinity towards cholesterol [20], [21] and that their upregulation is associated with suppression of apoptosis [22]. Supplementation of sphingolipids has been proposed to protect mouse oocytes from undergoing apoptosis [23], [24]. Whether the observed reduction in sphingomyelins in vitrified oocytes is linked to the modulation of cellular signaling and death pathways requires further investigation.

We examined the lipidomic profiles of oocytes that had not been vitrified, but were treated with the vitrification and warming solutions. We did not observe any significant effect of solutions on the phospholipid mass spectra (Fig. S2). However, as shown in Fig. 1, the distribution of fatty acid-enriched subcellular structures and membrane were altered in response to the vitrification solution treatment. Thus, while the vitrification solution-associated osmotic changes may temporarily alter the organization of fatty acid-enriched subcellular structures, it does not appear to cause quantifiable fluctuations in the phospholipid pool.

The negative effects associated with the duration of cryostorage on survival and fertilization rates seem to differ among species. Riggs and others report that cryostorage duration does not affect survival and pregnancy outcomes in humans [25]. In contrast, a longer duration of cryostorage for mouse oocytes leads to significant reductions in the rates of oocyte survival, fertilization, and embryonic development [26]. Therefore, phospholipid analyses in different species prior to and following cryostorage might provide insight into the association of phospholipids and developmental potential. Furthermore, it highlights the importance of preserving phospholipids during cryostorage.

In recent years, several approaches have been employed to assess the quality of oocytes after cryopreservation, focusing on total profiling of molecular components prior to and following the freezing process. Global gene expression profiling of oocytes in several species has mostly shown a negative influence of cryopreservation on the expression of a subset of genes [27]. A proteomic profiling study using mouse MII oocytes revealed a negative influence of 1,2-propanediol during slow freezing [28]. We propose that lipidomic approaches can be considered as a method to scrutinize the impact of media constituents and freezing methods on the quality of oocytes after cryostorage. Whether a reduction in the amount of certain phospholipid(s) has specific functional implications in oocyte biology requires further investigation. Such work will show how the identified phospholipids can be used as biomarkers for oocytes undergoing vitrification. It will also be interesting to ascertain whether slow freezing has a different effect on phospholipid profile. Such studies will provide insightful information as to which cryopreservation method is more effective in preserving lipids during cryostorage and help devise an improved condition for lipid preservation if necessary.

Abundant phospholipids that can be easily detected and assigned make ideal biomarkers of oocyte undergoing vitrification. The phospholipids that showed significant reduction after vitrification, as presented in Fig. 6C, can be used as ideal biomarkers for healthy oocytes after vitrification. Furthermore, these phospholipids can serve as a basis of comparison in studies investigating the influence of different cryopreservation conditions.

MALDI-MS analysis of phospholipids has been used widely to compare the lipid profile in normal and cancer tissues [29]–[31]. In reproduction, similar methods have been used to compare phospholipid profiles of oocytes and embryos of several mammalian species [12]–[14], [32], [33]. Further investigation into the effect of cryopreservation on the phospholipid profile of oocytes and embryos in various species will generate insightful information regarding the biological importance of lipid conservation during this process.

## Materials and Methods

### Oocyte collection

Mice were maintained in accordance with the policies of the Konkuk University Institutional Animal Care and Use Committee (IACUC). This study was approved by the Konkuk University IACUC (approval number KU12081). Four-week-old ICR mice were purchased from Orient-Bio (Gyeonggi-do, Korea) and were housed in a controlled barrier facility at Konkuk University. The mouse room is equipped with an automated light-dark cycle system and mice were fed a standard rodent chow (LabDiet, St. Louis, MO, USA). Mice were superovulated by injecting 7.5 IU PMSG (Sigma-Aldrich, St. Louis, MO, USA) and 7.5 IU hCG at a 48 h intervals. After 13–14 h after hCG injection, mice were sacrificed and oviducts were collected. Each mouse typically ovulated approximately 17–20 cumulus-oocyte complexes (COCs). COCs were collected by oviduct flushing 13–14 h post-hCG injection. Cumulus cells were removed by treating COCs with hyaluronidase (300 µg/ml) for 2 min. MII oocytes were collected, transferred to Quinn’s Advantage medium with *N*-2-hydroxyethylpiperazine-*N′*-2-ethanesulfonic acid (HEPES, Cooper Surgical, Trumbull, CT, USA) containing 20% fetal bovine serum (FBS, Gibco, Life Technologies, Grand Island, NY, USA), and cultured at 37°C.

### Vitrification and warming of mouse oocytes

Vitrification solutions contained EG (Sigma-Aldrich) and DMSO (Sigma-Aldrich) as cryoprotectants [34], [35]. MII oocytes from 20–25 mice were pooled and pre-equilibrated in an equilibration solution (EQ) containing 7.5% EG, 7.5% DMSO, and 20% FBS in phosphate-buffered saline (PBS) [34]. Oocytes were then transferred to the vitrification solution containing 15% EG, 15% DMSO, 20% FBS, and 0.5 M sucrose in PBS (Fisher Scientific, St. Louis, MO, USA). After 20 s, 20 oocytes were loaded onto an electron microscopy copper grid (Ted Pella, Inc., Redding, CA, USA) and submerged in liquid nitrogen (LN_2_). Vitrified oocytes were stored in LN_2_ for 2 weeks. To thaw the oocytes, the grids were taken out of the LN_2_ and directly put into thawing media (0.5 M sucrose and 20% FBS in PBS) for 2.5 min. Thawed oocytes (n = 100) were collected and sequentially incubated for 2.5 min in solutions containing decreasing concentrations of sucrose (0.25 M, 0.125 M, and 0 M). Finally, oocytes recovered for 1 h in Quinn’s-HEPES media containing 20% FBS and incubated at 37°C in 5% CO_2_. Oocytes without obvious morphological deformation or discoloration were selected and used for further analyses. Oocytes that survived the thawing process (∼98%) were subjected to lipid extraction. In our routine laboratory procedures, vitrified-warmed mouse oocytes showed average of 62% fertilization rate and ∼50% developmental rate to the blastocyst stage [36].

### Fluorescence staining and confocal live imaging

BODIPY fatty acid 500/510 (D-3823) and CellMask Deep Red Plasma Membrane Stain (C10046) were purchased from Invitrogen (Carlsbad, CA, USA). BODIPY was dissolved in DMSO and diluted to 10 µg/ml in Quinn’s-HEPES media (Cooper Surgical). CellMask was used at 2.5 µg/ml in media. MII oocytes were stained with the fluorescent dyes for 30 min, washed three times with media, and transferred to a glass-bottom confocal dish (SPL Life Sciences, Pocheon, Korea) [37], [38]. Live images were captured using a Zeiss LSM710 confocal microscope (Zeiss, Germany) outfitted with a 40×C-Apochromat water immersion objective.

### Reagents

HPLC-grade methanol, chloroform, and water were purchased from Burdick and Jackson (Muskegon, MI, USA). 2,5-dihydroxybenzoic acid (DHB), α-cyano-4-hydroxycinnamic acid (CHCA), 9-aminoacridine, and trifluoroacetic acid (TFA) were purchased from Sigma-Aldrich.

### Phospholipid extraction

Phospholipids were extracted from MII oocytes (100 oocytes/group) by using the Bligh & Dyer method [39]. The following three oocyte groups were prepared: fresh MII oocytes, oocytes treated with equilibration and vitrification solutions only (solution-treated), and oocytes vitrified for 2 weeks. As shown in Fig. 1A, two sets of vitrified-warmed oocytes with matching controls (represented as Set 1 and Set 2 in the diagram) were used. Since phospholipid extraction and subsequent analyses were all performed on the day of oocyte preparation, control groups were prepared on the day of thawing vitrified oocytes of the previous set. Therefore, another set of control oocytes were prepared when extracting phospholipids from vitrified-warmed oocytes of the 2^nd^ set (Fig. 1A). For the extraction, oocytes were first rinsed with PBS several times to remove traces of culture media and then transferred into 3 ml of CHCl_3_:methanol (1∶2, v/v) in 15 ml conical glass tubes. Each sample was vortexed vigorously for 1 min, sonicated for 10 min, and cooled on ice for 10 min. After adding 2.3 ml of CHCl_3_:water (1∶1.3, v/v), the samples were vortexed vigorously and centrifuged at 2500×*g* for 10 min. The bottom organic phase containing the lipids was dried using a speed vacuum. All experiments were performed in duplicate.

### Lipid MALDI-TOF MS analysis

A binary matrix solution was prepared by dissolving 3.5 mg each of DHB and CHCA into 1 ml of 70% methanol containing 0.1% TFA for the positive mode lipid MALDI-MS analysis [29], [30], [40], [41]. 9-aminoacridine (5 mg/ml; dissolved in 6∶4 isopropanol: acetonitrile, v/v) was used for the negative mode analysis [40], [42]. The matrix suspensions (1.5 µl) were pipetted onto a stainless steel 384-well target plates (Bruker Daltonics, Billerica, MA, USA) and then dried in vacuum desiccator to equilibrate the sample and matrix and reduce variability in the spectra. After sample spotting, each spot was analyzed directly by MS. MALDI MS analysis was performed using an Ultraflex III TOF/TOF mass spectrometer (Bruker Daltonics) equipped with a 200-Hz smart beam laser as the ionization source. The spectra were acquired using the following perameters: delay, 180 ns; ion source 1, 25 kV; ion source 2, 21.65 kV; and lens voltage, 9.2 kV. The MALDI-TOF was optimized for 550–1100 Da molecules and an average of 1000 shots/spot was acquired. Before each data acquisition, an external calibration was conducted using lipid-mixed calibration standards with mass-to-charge (m/z) ranges of 510–810 Da (positive ion mode) and 564–906 Da (negative ion mode). MALDI LIFT analysis was performed directly on the sample spot after the MALDI-MS analysis [43]. LIFT data were annotated using the lipid database, Lipidomics Gateway (www.lipidmaps.org).

### Preprocessing of MALDI MS data

For each group, spectra were obtained 10 and 7 times in the positive and negative mode, respectively. All preprocessing steps were performed using a MALDIquant R package [44]. The intensity in single spectra were transformed to a square root scale for variance stabilization and smoothed using a moving average algorithm. The spectrum background was evaluated using the statistics-sensitive non-linear iterative peak-clipping (SNIP) algorithm and used for baseline correction. The intensities of multiple spectra were normalized using the probabilistic quotient normalization method. The features with signal-to-noise ratios higher than 5 were detected as peaks. The peaks affiliated with the same mass were aligned by the statistical regression-based approach using the identification of landmark peaks and the estimation of a non-linear warping function. The raw data showing peak intensities are included in the Supporting Information (Tables S1–S3).

### Statistical analysis of MALDI MS data

To compare multiple spectra from different samples, the following statistical analyses were performed using MetaboAnalyst 2.0 [45], a web-based software for quantitative data analysis. The input data for MetaboAnalyst analyses are given in the Supporting Information (Tables S4–S6). Half of the minimum positive value in the data replaced missing values in the data obtained from the preprocessing procedure. The intensity values of each peak across multiple spectra were mean-centered and divided by the standard deviation. Principal component analysis (PCA) was performed to show the relationship between variance in the data and differences among fresh and 2-week vitrified oocyte samples. The differentially expressed phospholipids were identified in volcano plots comparing fresh and vitrified oocytes by using the following criteria: (1) *P* values from the *t*-tests were less than 0.01 and (2) absolute fold changes were greater than 2. The raw data of volcano plots are given in the Supporting Information (Tables S7&S8). To show the relationship between samples and features, hierarchical clustering of the differentially expressed phospholipids was performed using Euclidean distances and Ward linkage. The Pearson’s correlation coefficient of the features were calculated and clustered to identify correlating peaks.

## Supporting Information

Figure S1 **Pure matrix profiles of (A) binary matrix (positive ion mode) and (B) 9-aminoacridine (negative ion mode).**
(DOC)

Figure S2 (A) Average mass spectra for lipids that are positive in fresh oocytes (red) and solution-treated oocytes (green). (B) A principal component analysis plot for phospholipid mass spectrum of fresh oocytes (red), solution-treated control oocytes (green), and 2-weeks vitrified oocytes (blue).
(DOC)

Figure S3 **Annotation of differentially expressed phospholipids (** **Table 1** **) by using the LIFT technique.**
(PDF)

Table S1 **MALDIquant output file: fresh vs vitrified oocytes in positive mode.**
(XLSX)

Table S2 **MALDIquant output file: fresh vs vitrified oocytes in negative mode.**
(XLSX)

Table S3 **MALDIquant output file: fresh vs media-treated vs vitrified oocytes in positive mode.**
(XLSX)

Table S4 **Metaboanalyst input file: fresh vs vitrified oocytes in positive mode.**
(XLSX)

Table S5 **Metaboanalyst input file: fresh vs vitrified oocytes in negative mode.**
(XLSX)

Table S6 **Metaboanalyst input file: fresh vs media-treated vs vitrified oocytes in positive mode.**
(XLSX)

Table S7 **Volcano plot data: fresh vs vitrified oocytes in positive mode.**
(XLSX)

Table S8 **Volcano plot data: fresh vs vitrified oocytes in negative mode.**
(XLSX)
