# Supplementary material for: Analysis of the Phospholipid Profile of Metaphase II Mouse Oocytes Undergoing Vitrification
Source: PLoS One. 2014 Jul 17;9(7):e102620. doi: 10.1371/journal.pone.0102620 (PMC4102530; doi:10.1371/journal.pone.0102620)
Supplement: Figure S1 — Pure matrix profiles of (A) binary matrix (positive ion mode) and (B) 9-aminoacridine (negative ion mode). (DOC) [file pone.0102620.s001.doc]

Figure S1. Pure matrix profiles of (A) binary matrix (positive ion mode) and (B) 9-aminoacridine (negative ion mode).


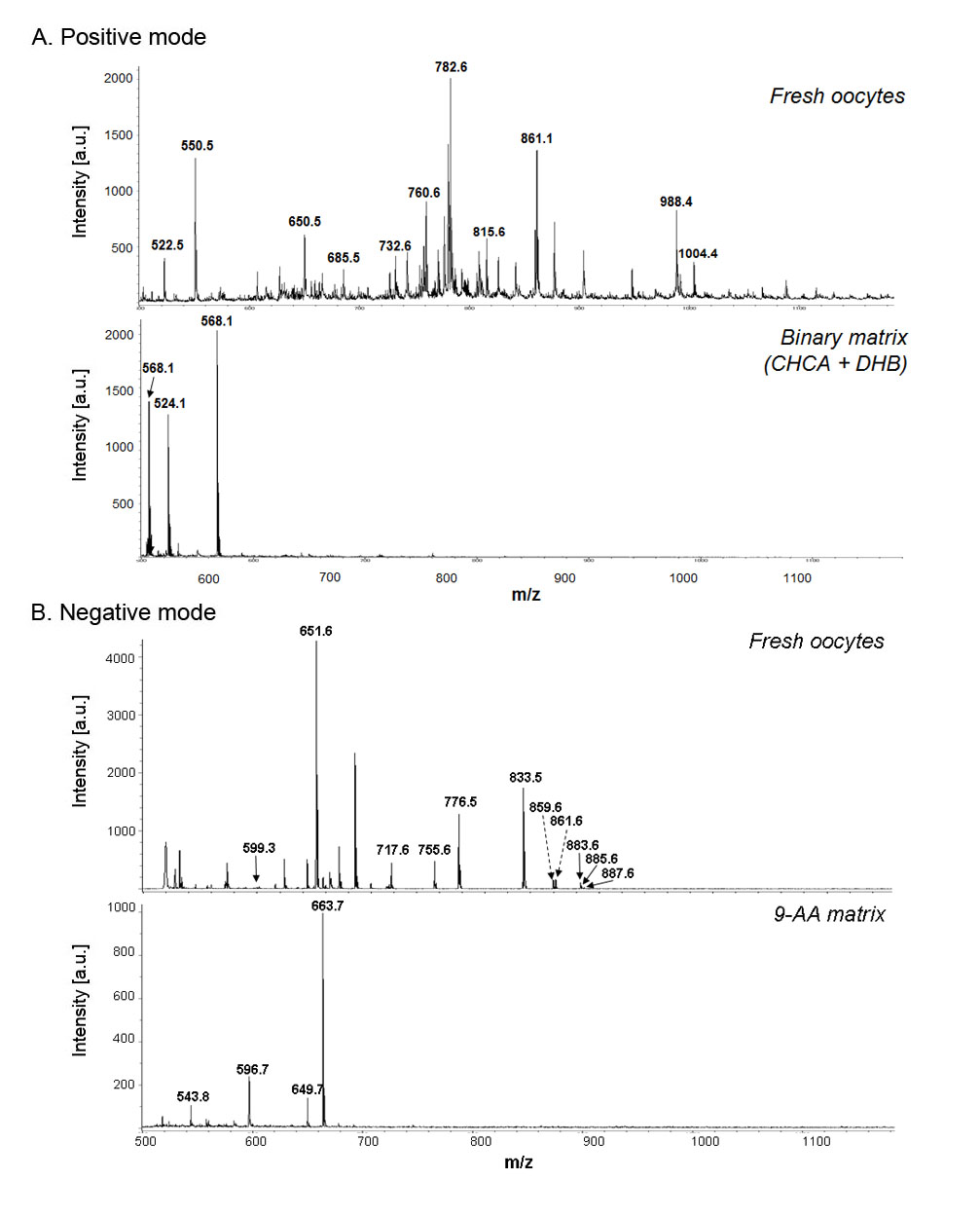


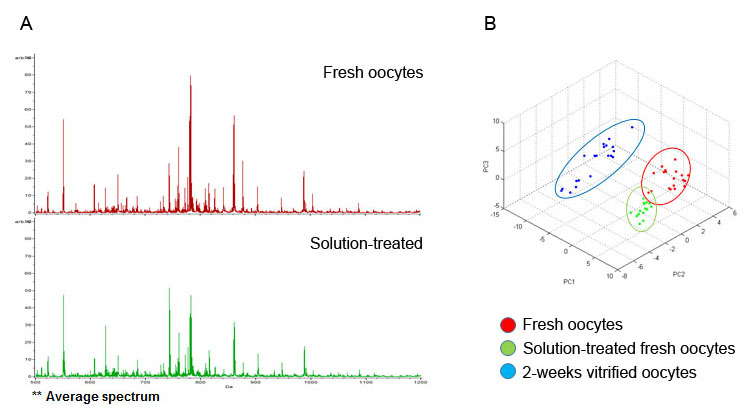


Figure S2.
