# Supplementary material for: Analysis of the Phospholipid Profile of Metaphase II Mouse Oocytes Undergoing Vitrification
Source: PLoS One. 2014 Jul 17;9(7):e102620. doi: 10.1371/journal.pone.0102620 (PMC4102530; doi:10.1371/journal.pone.0102620)

Figure S2.

(A) Average mass spectra for lipids that are positive in fresh oocytes (red) and solution-treated oocytes (green).

(B) A principal component analysis plot for phospholipid mass spectrum of fresh oocytes (red), solution-treated control oocytes (green), and 2-weeks vitrified oocytes (blue).


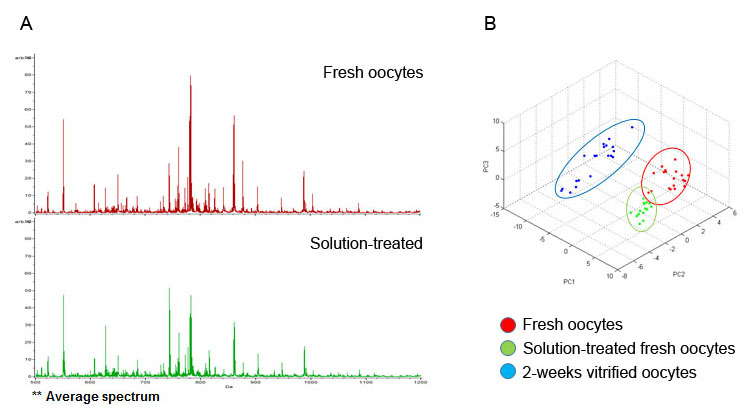

Supplement: Figure S2 — (A) Average mass spectra for lipids that are positive in fresh oocytes (red) and solution-treated oocytes (green). (B) A principal component analysis plot for phospholipid mass spectrum of fresh oocytes (red), solution-treated control oocytes (green), and 2-weeks vitrified oocytes (blue). (DOC) [file pone.0102620.s002.doc]
