# Supplementary material for: Analysis of the Phospholipid Profile of Metaphase II Mouse Oocytes Undergoing Vitrification
Source: PLoS One. 2014 Jul 17;9(7):e102620. doi: 10.1371/journal.pone.0102620 (PMC4102530; doi:10.1371/journal.pone.0102620)
Supplement: Figure S3 — Annotation of differentially expressed phospholipids ( Table 1 ) by using the LIFT technique. (PDF) [file pone.0102620.s003.pdf]

Figure S3. Annotation of differentially expressed phospholipids (Table 1) by using the LIFT technique.

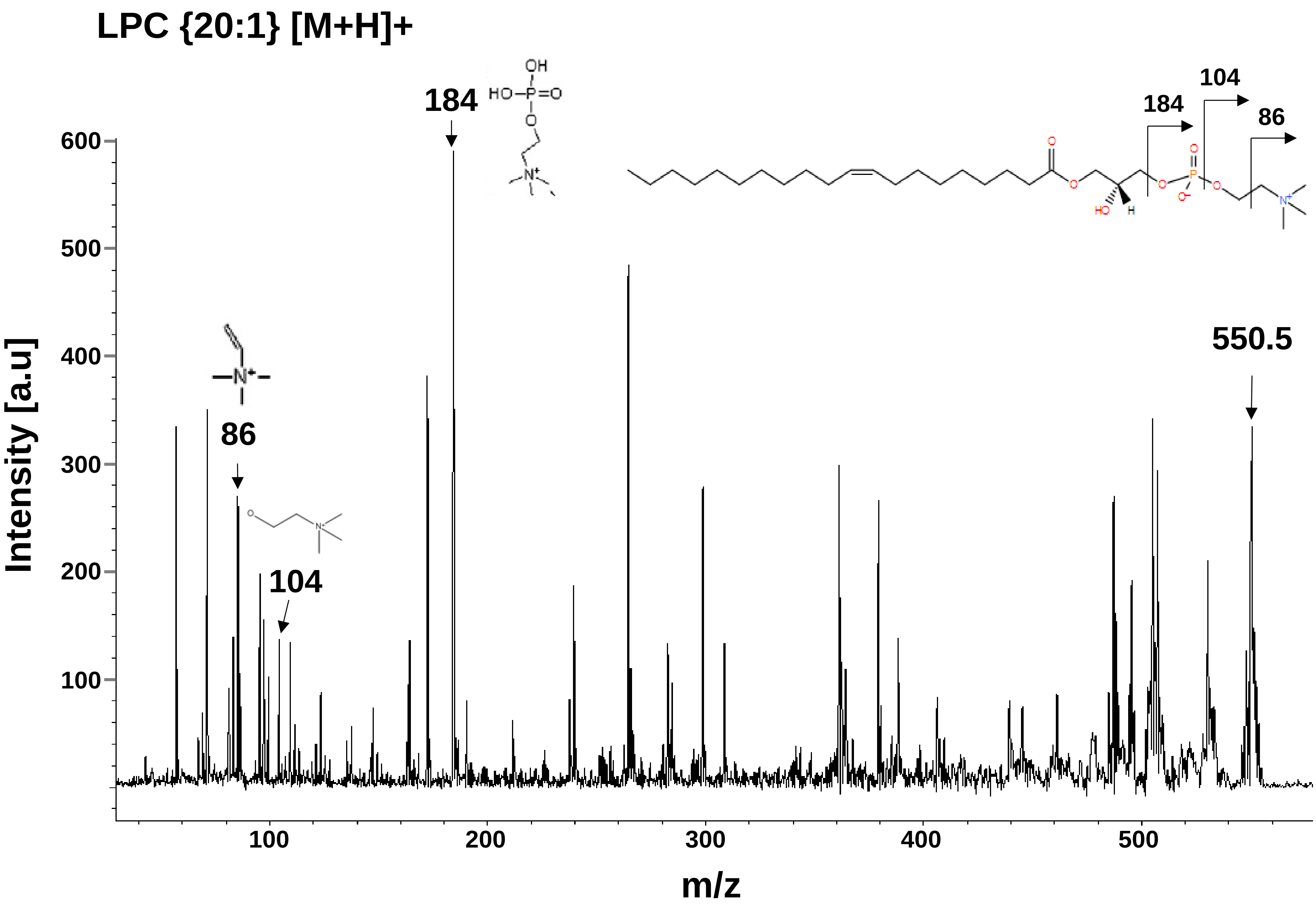

LPC {20:1} [M+H]+
Intensity [a.u]
m/z
184
86
104
550.5
600
500
400
300
200
100
100
200
300
400
500

PC {26:0} [M+H]+

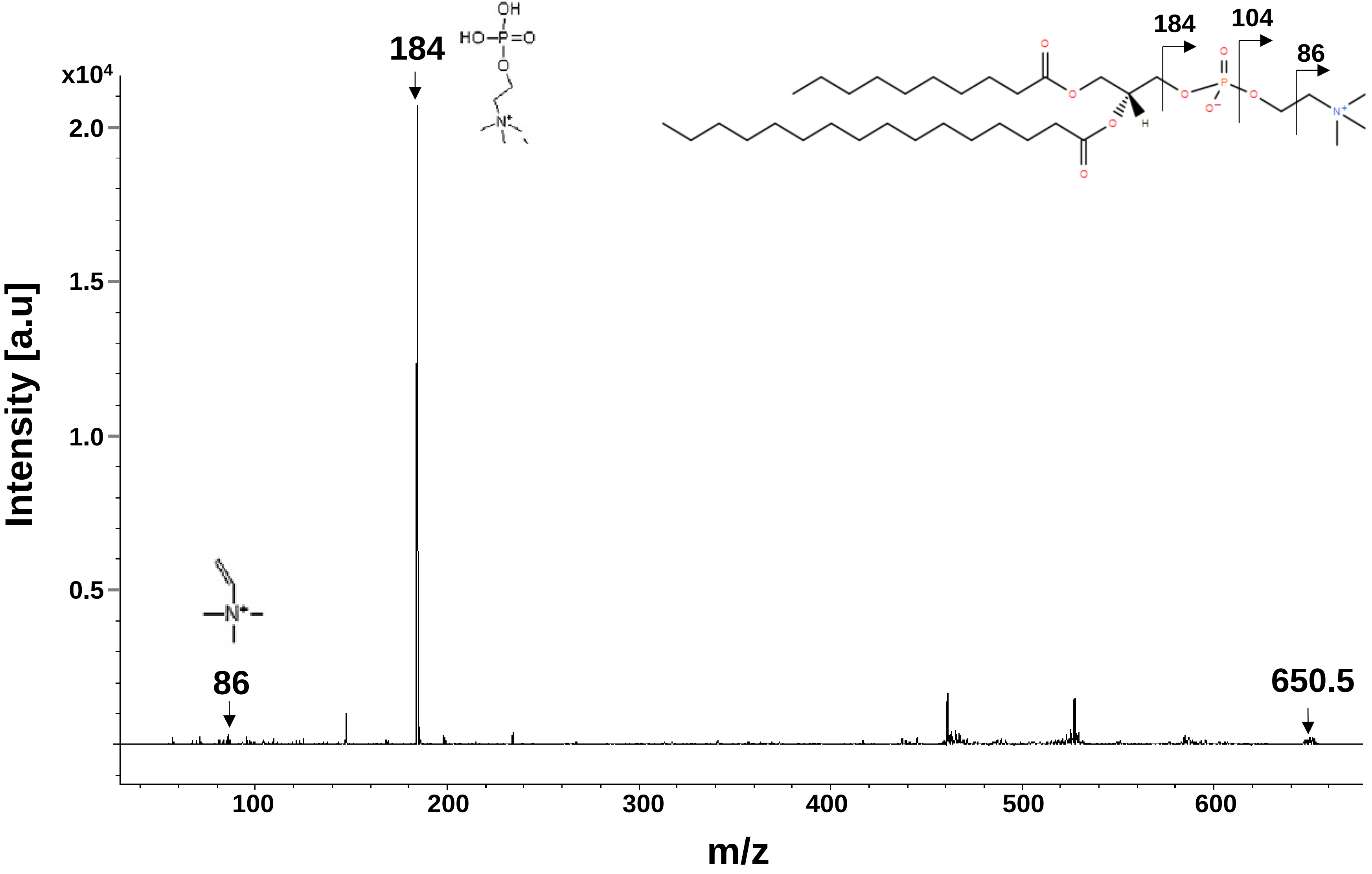

184
OH
HO–P=O
O
N+
184
104
86
O
O
O
O
O
P
O−
O
N+
H
O
x10^4
2.0
1.5
1.0
0.5
Intensity [a.u]
86
650.5
100
200
300
400
500
600
m/z

SM {d30:1} [M+K]+

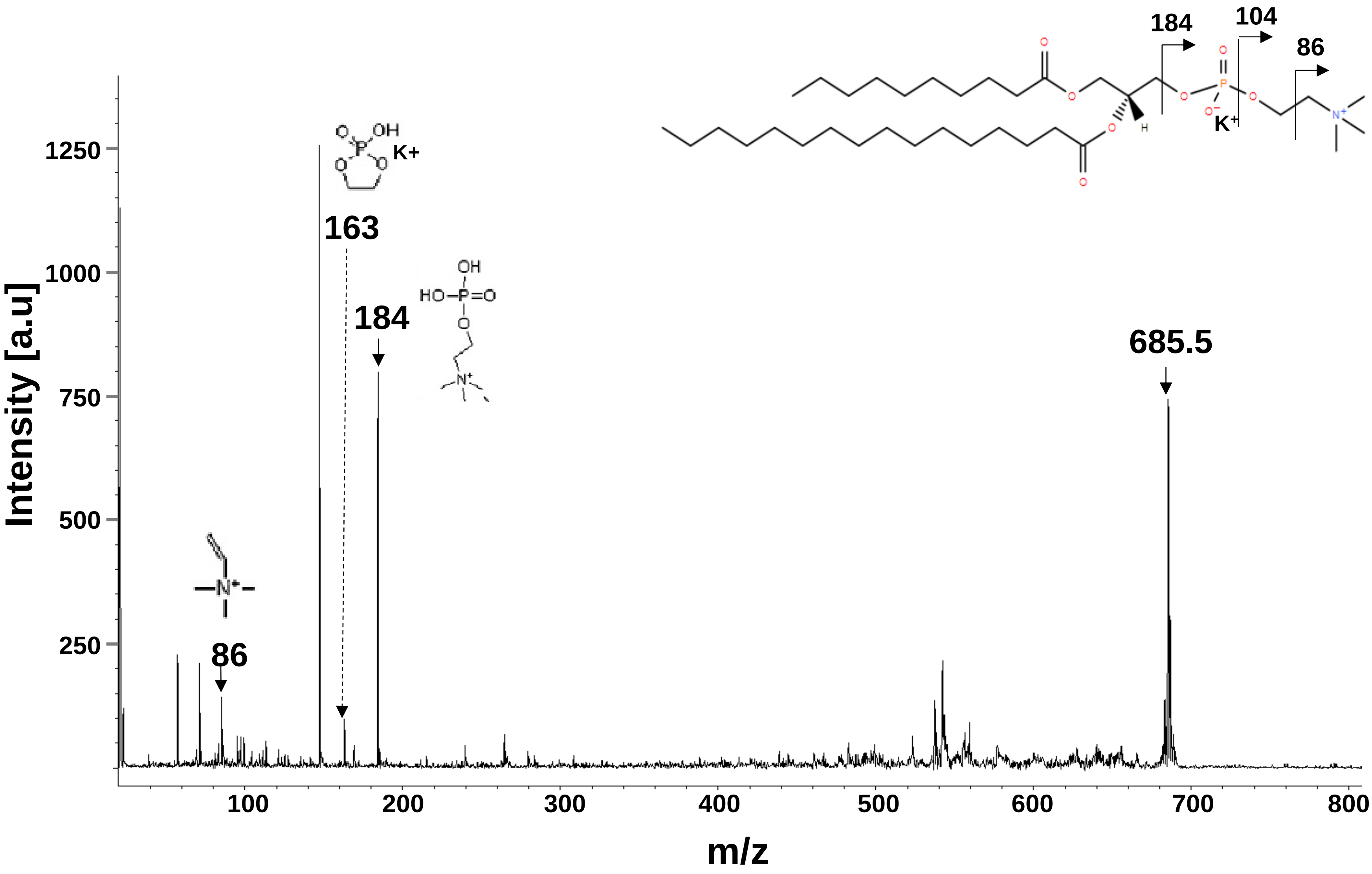

Intensity [a.u]
m/z
1250
1000
750
500
250
100
200
300
400
500
600
700
800
163
K+
184
86
685.5
184
104
86
K+

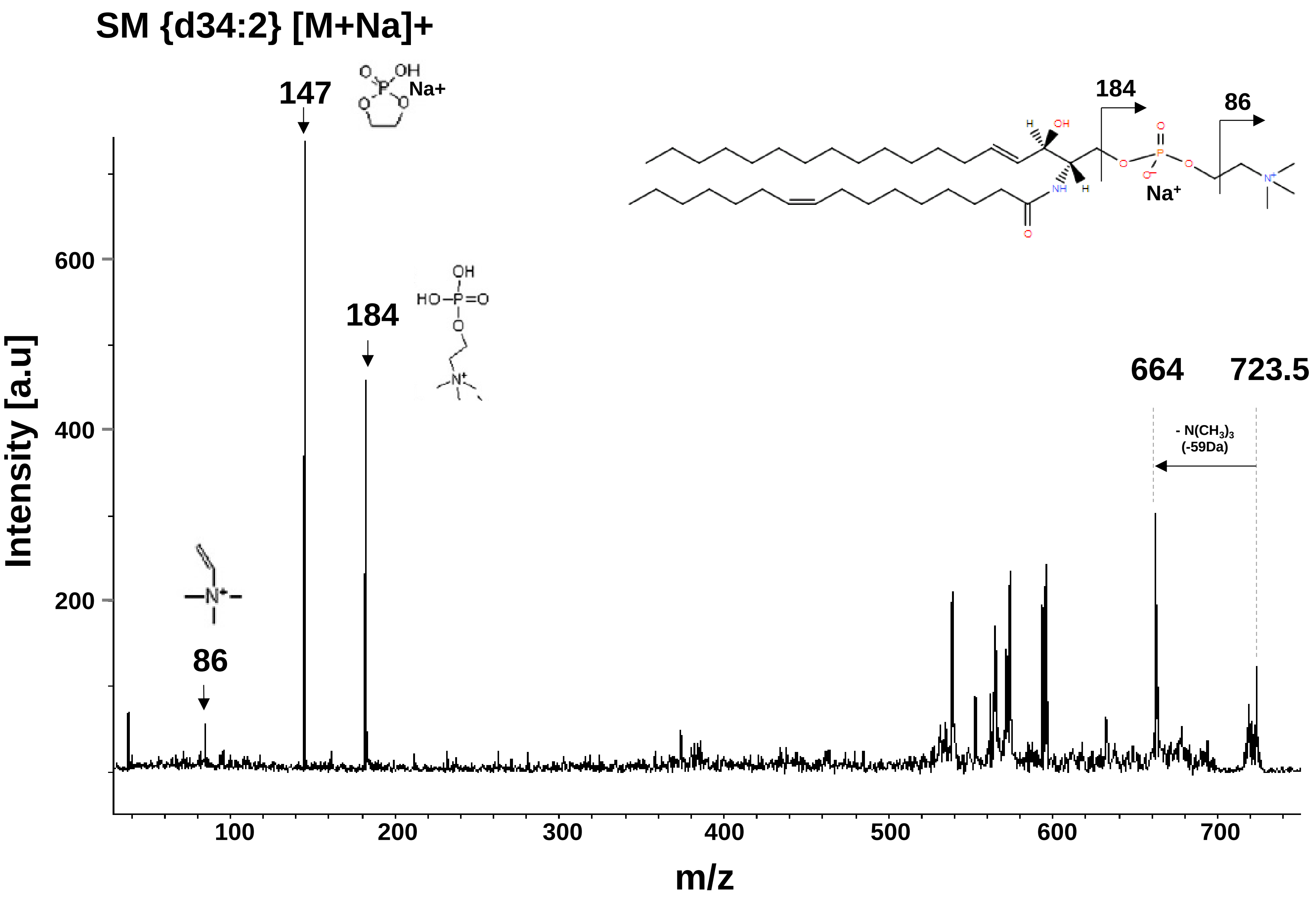
SM {d34:2} [M+Na]+
147
Na+
184
86
Na+
184
664
723.5
- N(CH3)3
(-59Da)
86
Intensity [a.u]
600
400
200
100
200
300
400
500
600
700
m/z

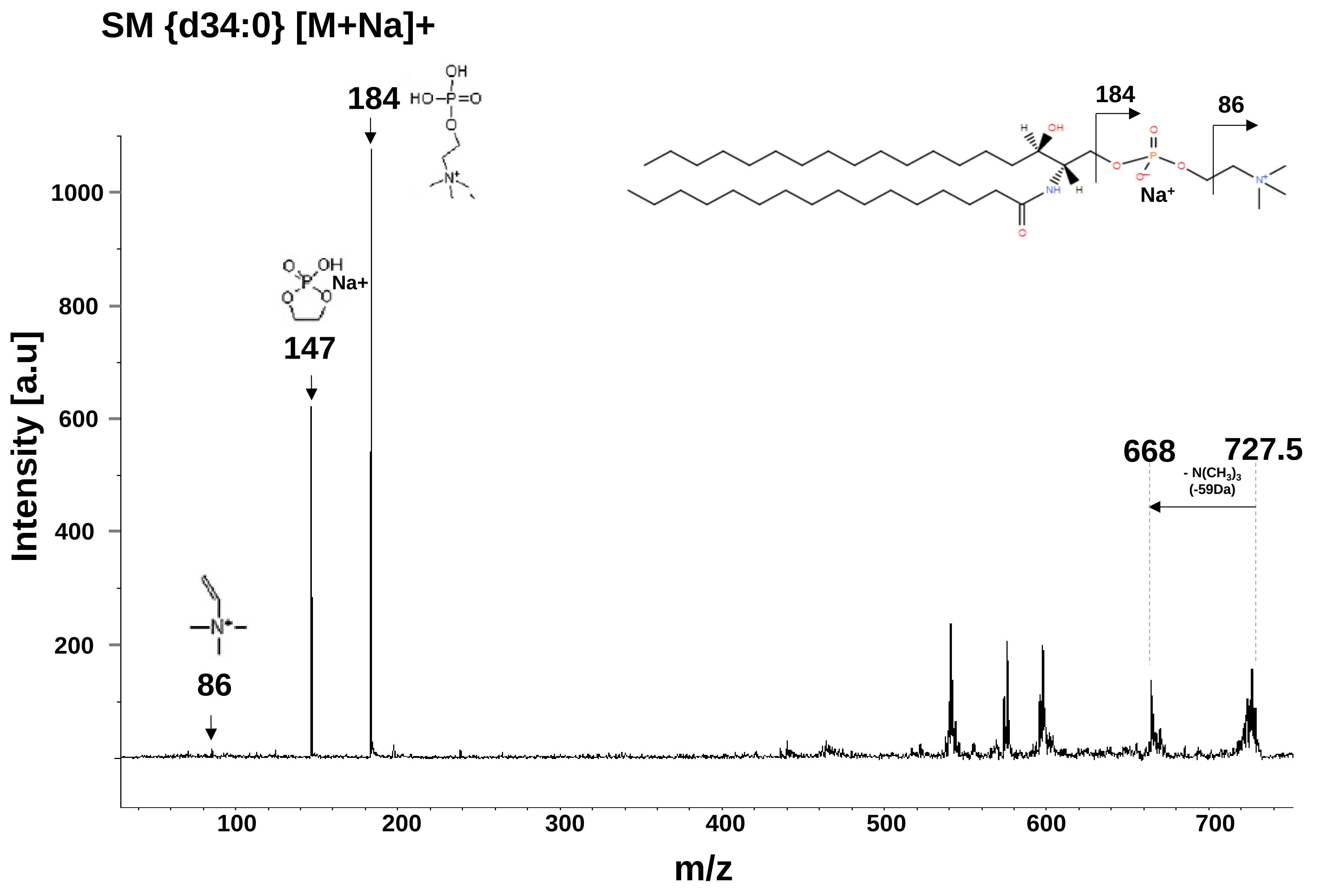

SM {d34:0} [M+Na]+
184
147
Na+
86
184
86
Na+
668
727.5
- N(CH3)3
(-59Da)
Intensity [a.u]
1000
800
600
400
200
100
200
300
400
500
600
700
m/z

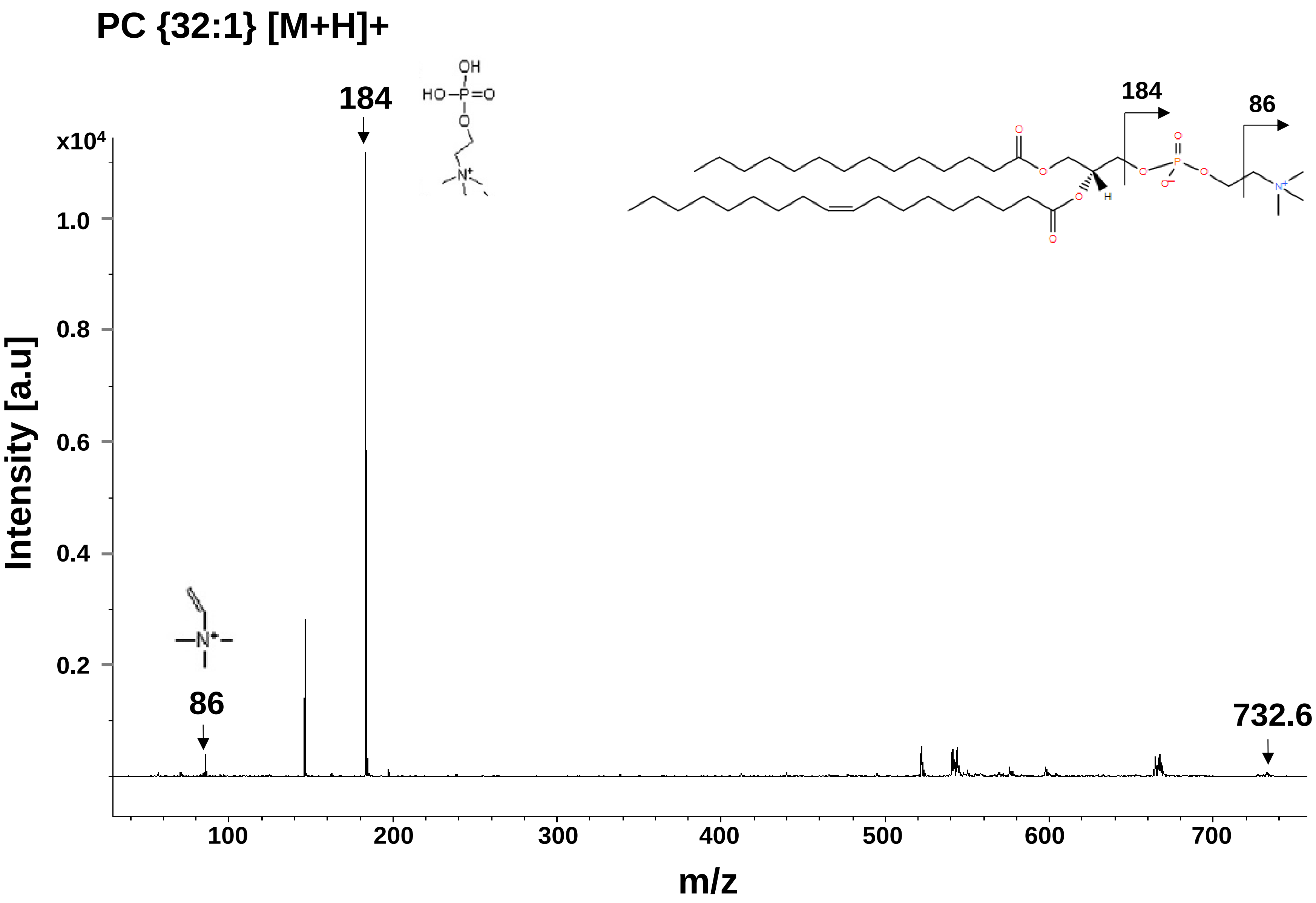

PC {32:1} [M+H]+
184
x10⁴
1.0
0.8
0.6
0.4
0.2
Intensity [a.u]
86
732.6
100
200
300
400
500
600
700
m/z
HO–P=O
OH
N+

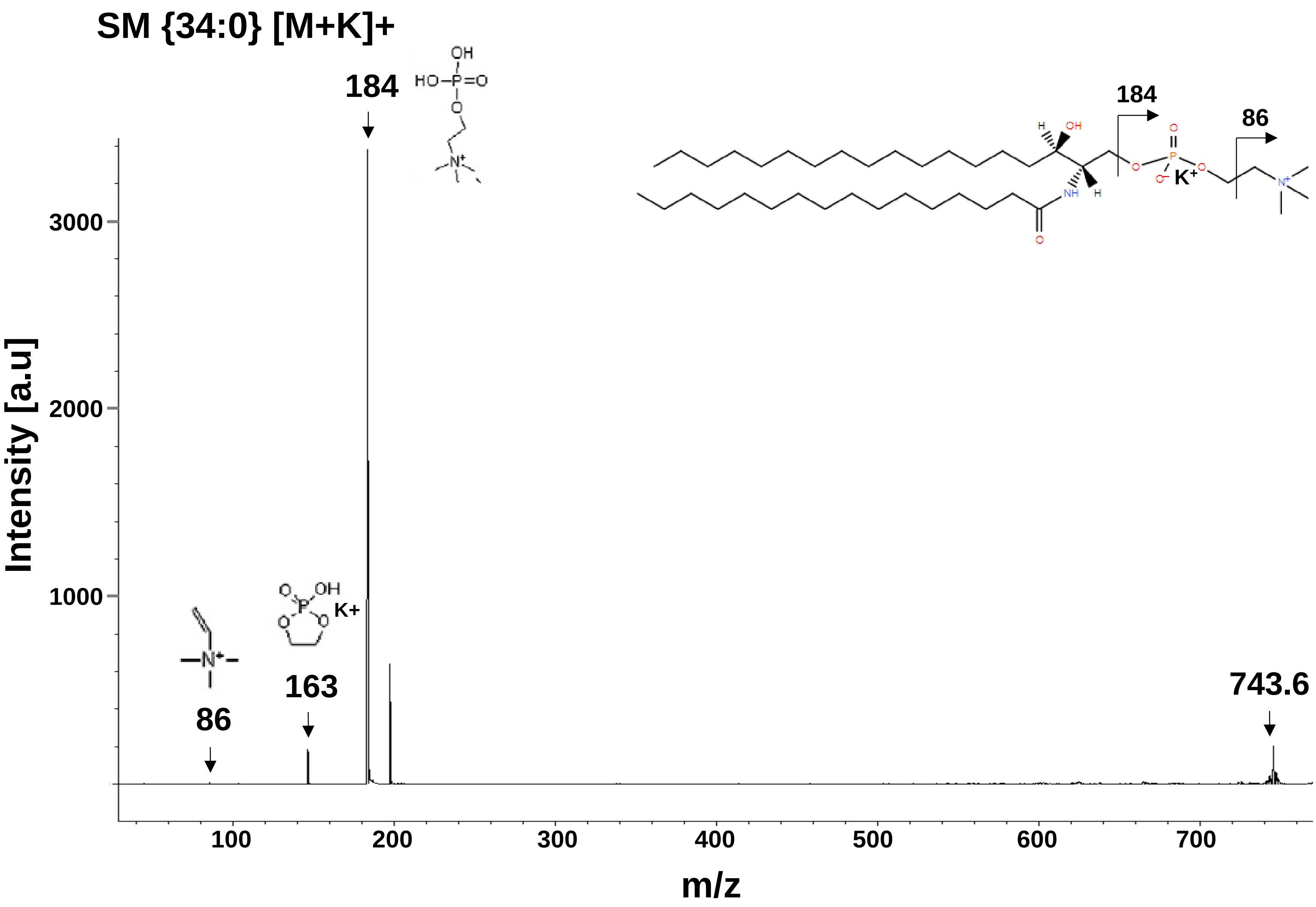

SM {34:0} [M+K]+
184
86
163
743.6
K+
Intensity [a.u]
m/z
1000
2000
3000
100
200
300
400
500
600
700

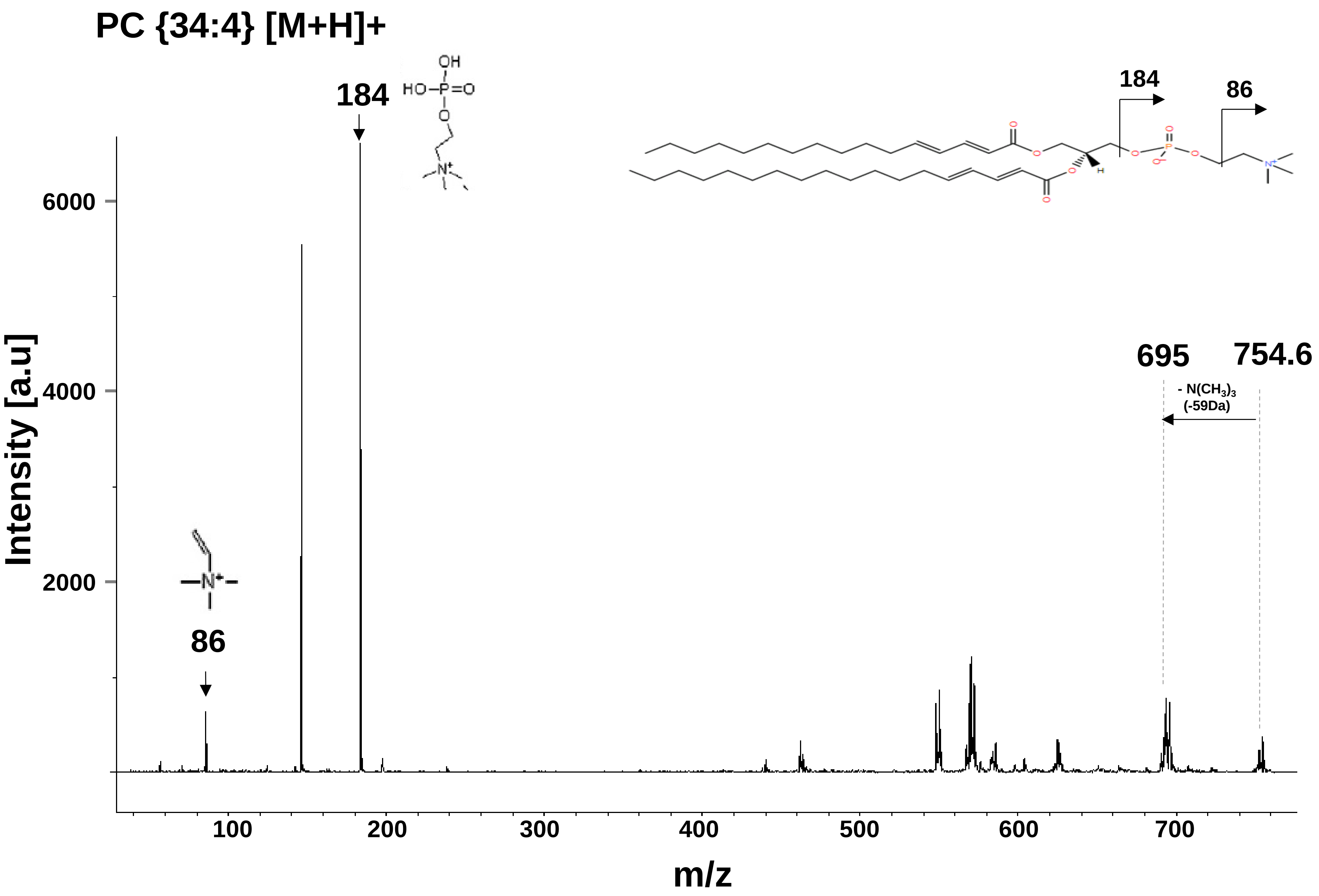

PC {34:4} [M+H]+
184
86
695
754.6
- N(CH3)3
(-59Da)
6000
4000
2000
Intensity [a.u]
100
200
300
400
500
600
700
m/z

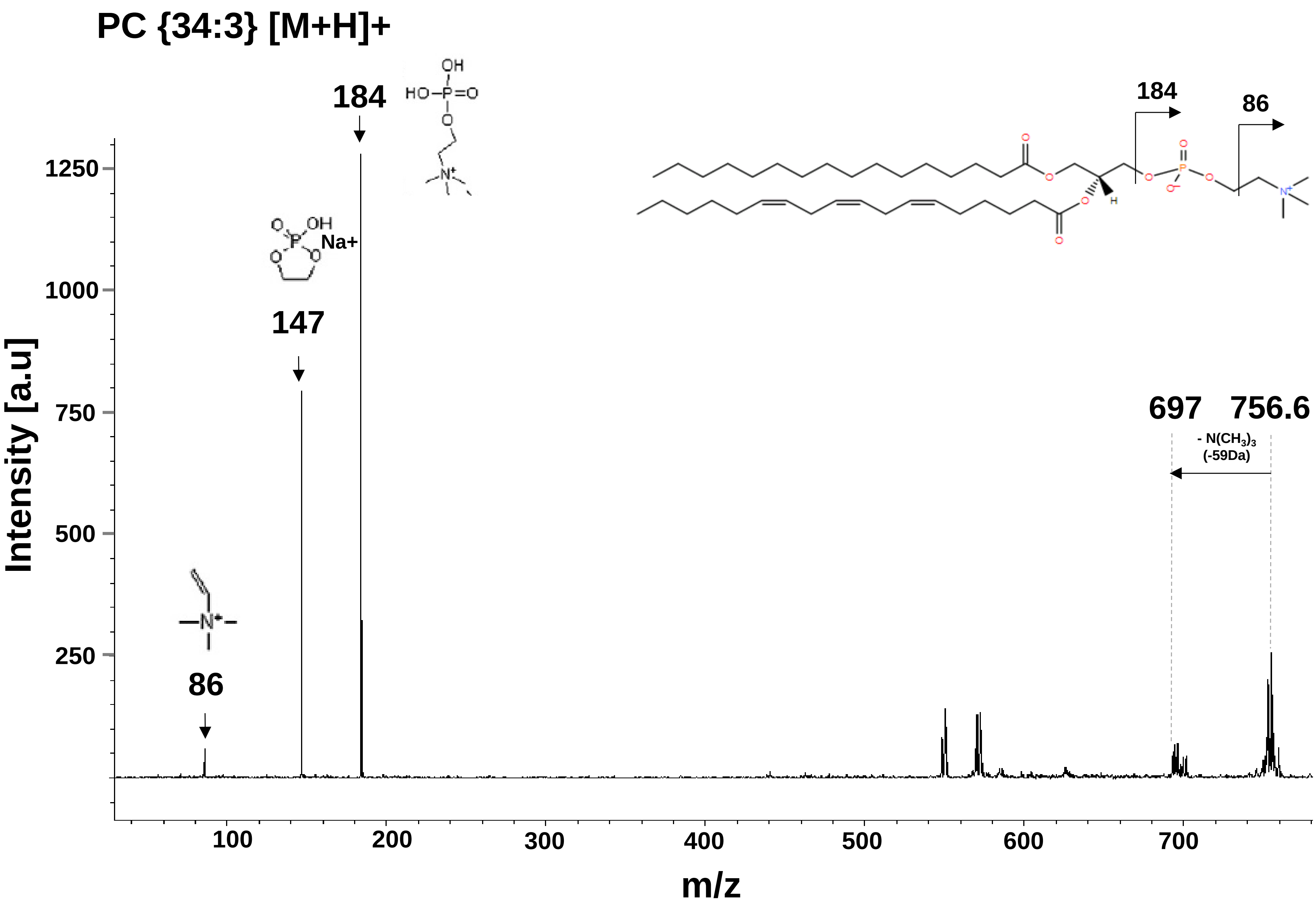

PC {34:3} [M+H]+
184
147
86
Na+
184
86
697
756.6
- N(CH3)3
(-59Da)
Intensity [a.u]
1250
1000
750
500
250
100
200
300
400
500
600
700
m/z

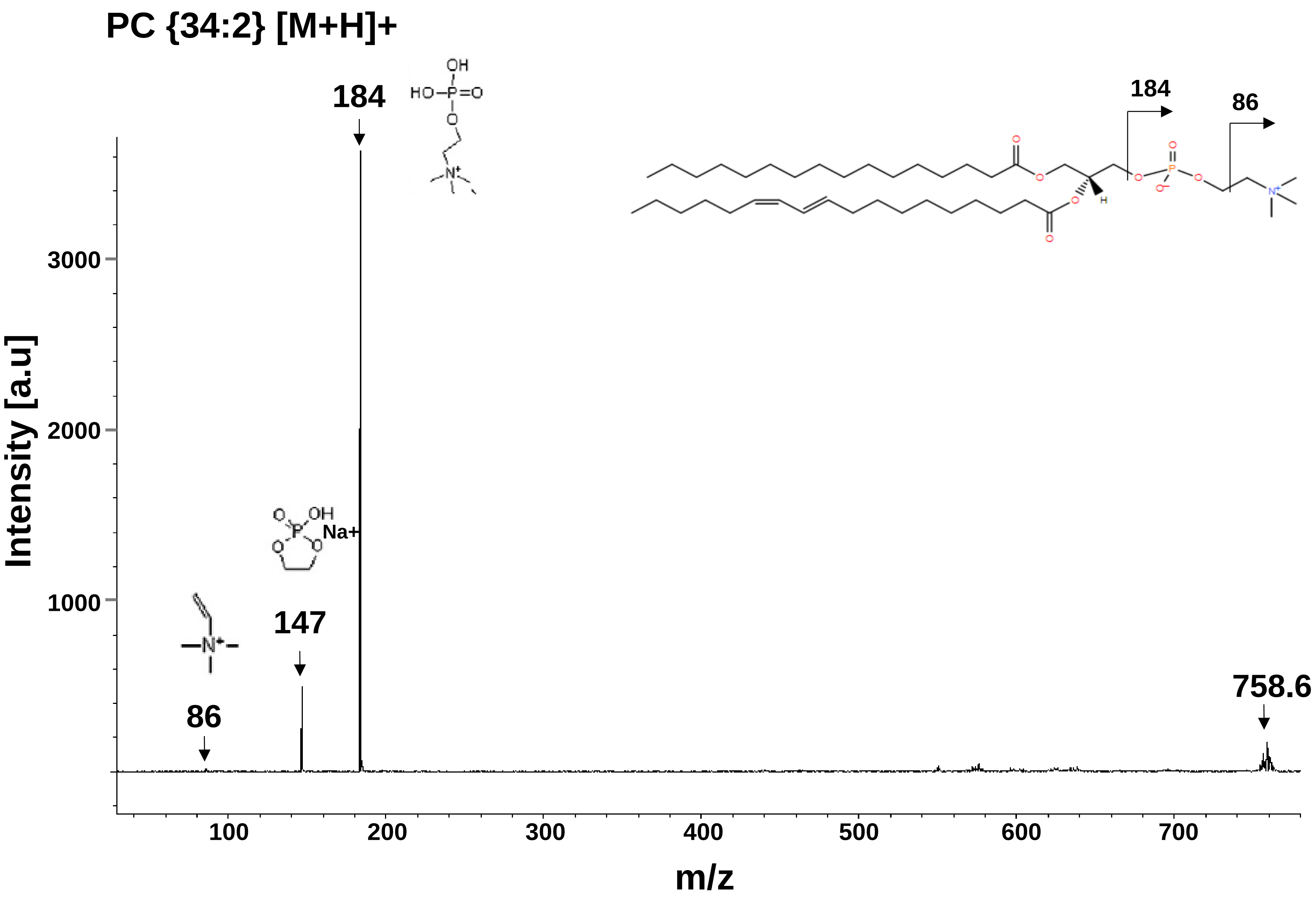
PC {34:2} [M+H]+
184
86
147
758.6
Na+
Intensity [a.u]
m/z
3000
2000
1000
100
200
300
400
500
600
700

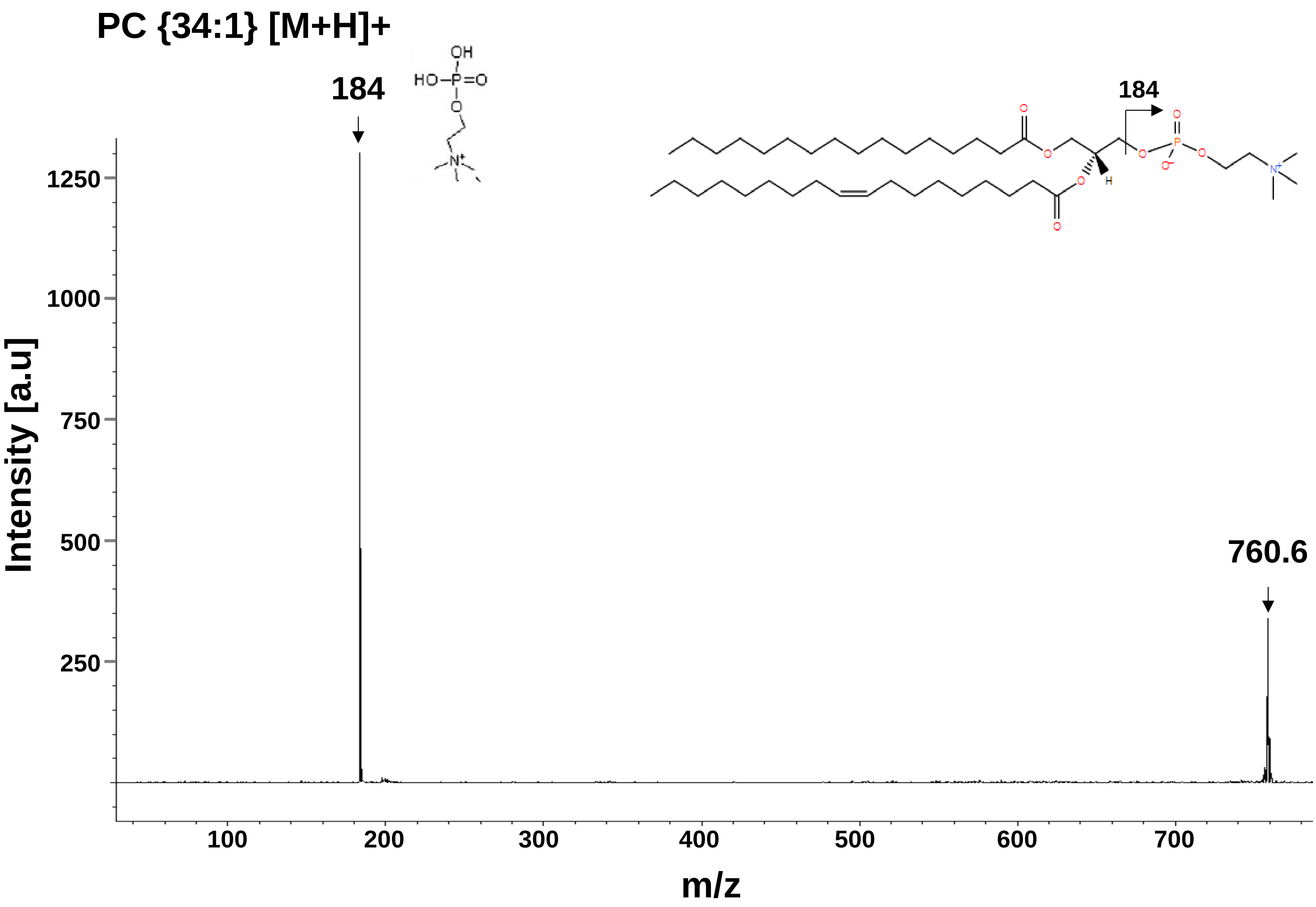

PC {34:1} [M+H]+
184
184
760.6
Intensity [a.u]
m/z
1250
1000
750
500
250
100
200
300
400
500
600
700

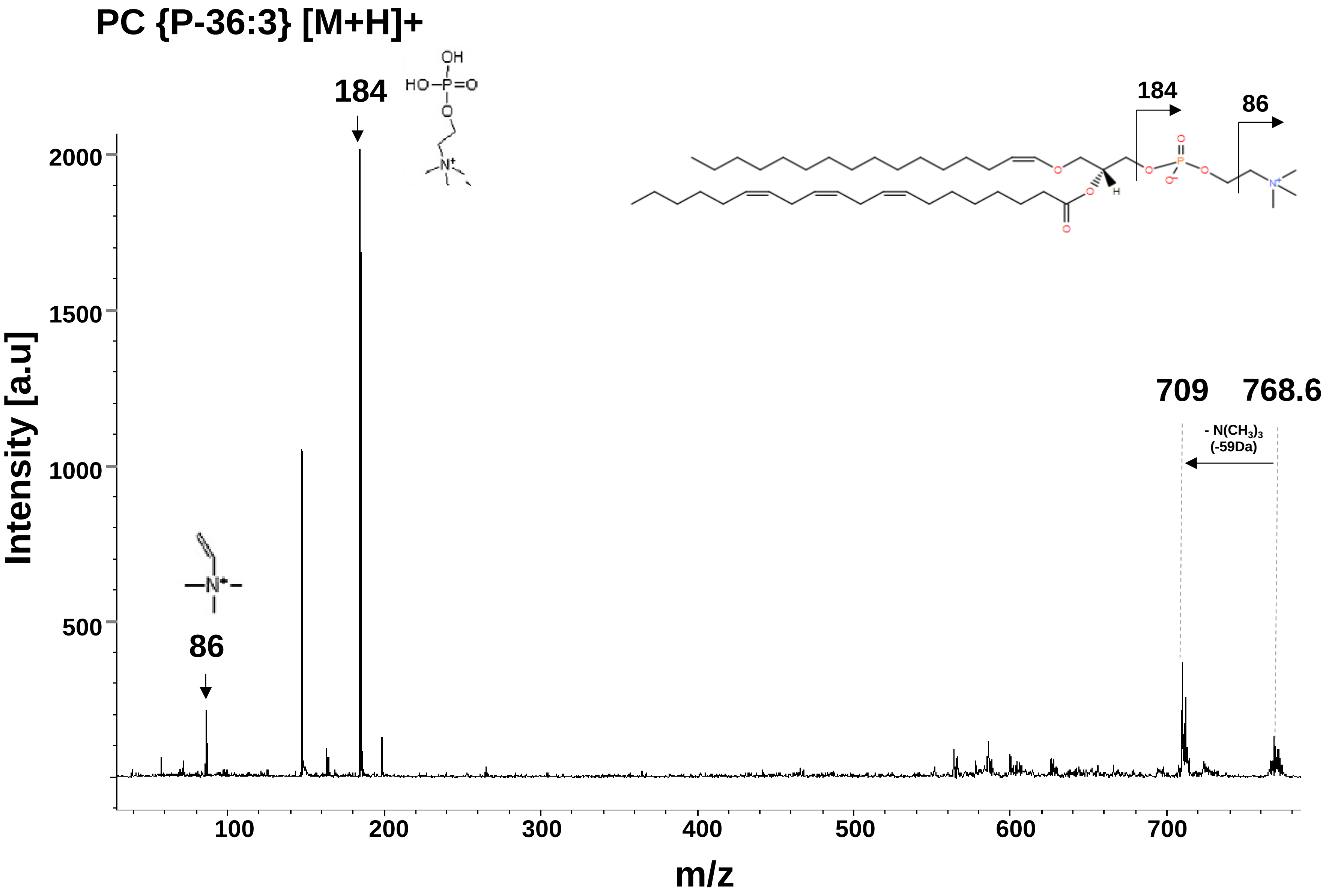

PC {P-36:3} [M+H]+
184
86
709
768.6
- N(CH3)3
(-59Da)
Intensity [a.u]
2000
1500
1000
500
100
200
300
400
500
600
700
m/z

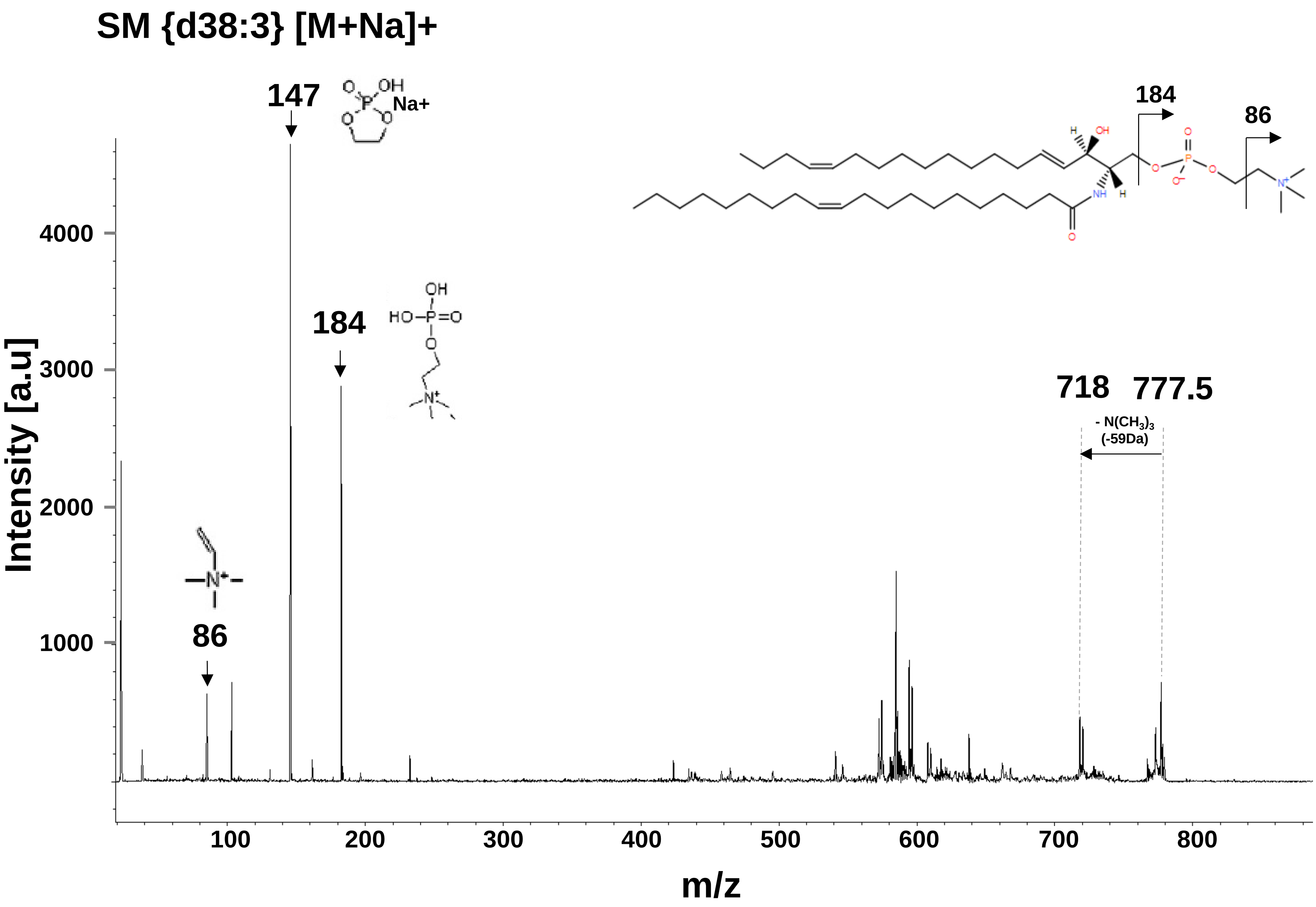

SM {d38:3} [M+Na]+
147
Na+
184
86
718
777.5
- N(CH3)3
(-59Da)
Intensity [a.u]
4000
3000
2000
1000
100
200
300
400
500
600
700
800
m/z

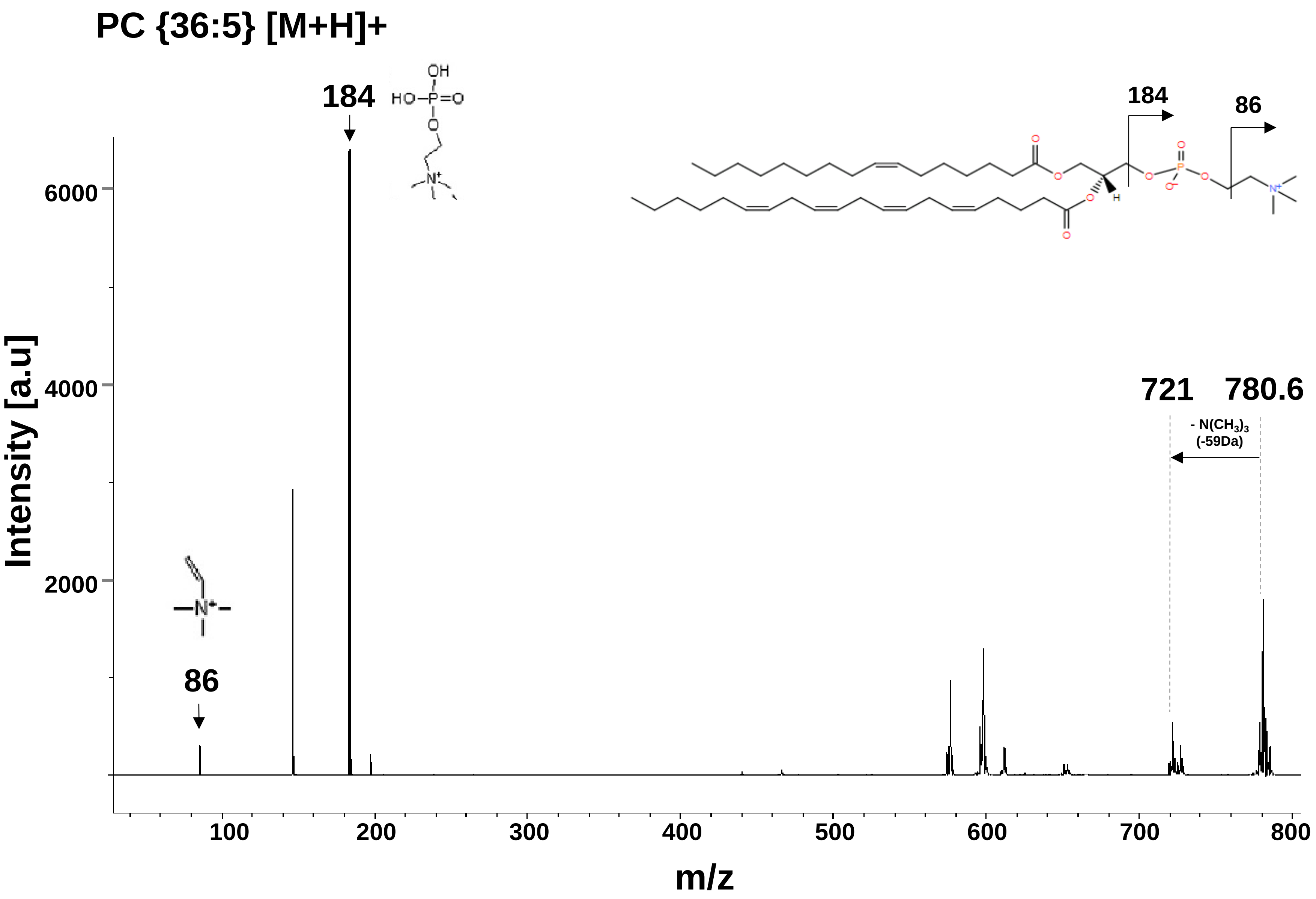
PC {36:5} [M+H]+
184
86
721
780.6
- N(CH3)3
(-59Da)
Intensity [a.u]
m/z
6000
4000
2000
100
200
300
400
500
600
700
800

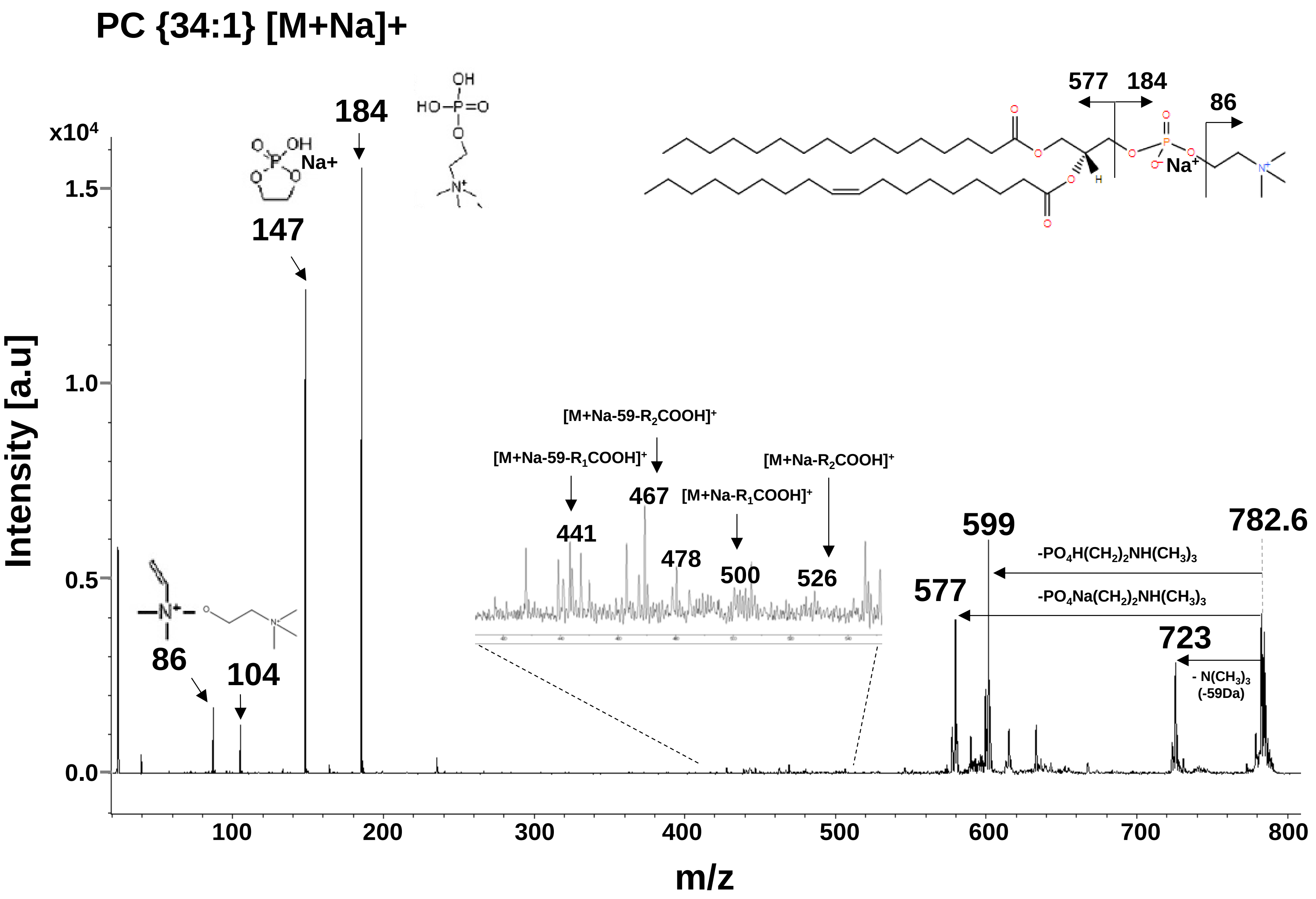
PC {34:1} [M+Na]+
x10^4
1.5
1.0
0.5
0.0
Intensity [a.u]
184
147
Na+
86
104
577 184
86
Na+
[M+Na-59-R2COOH]+
[M+Na-59-R1COOH]+
[M+Na-R2COOH]+
[M+Na-R1COOH]+
467
441
478
500
526
599
782.6
577
723
-PO4H(CH2)2NH(CH3)3
-PO4Na(CH2)2NH(CH3)3
- N(CH3)3
(-59Da)
100
200
300
400
500
600
700
800
m/z

PC {36:2} [M+H]+

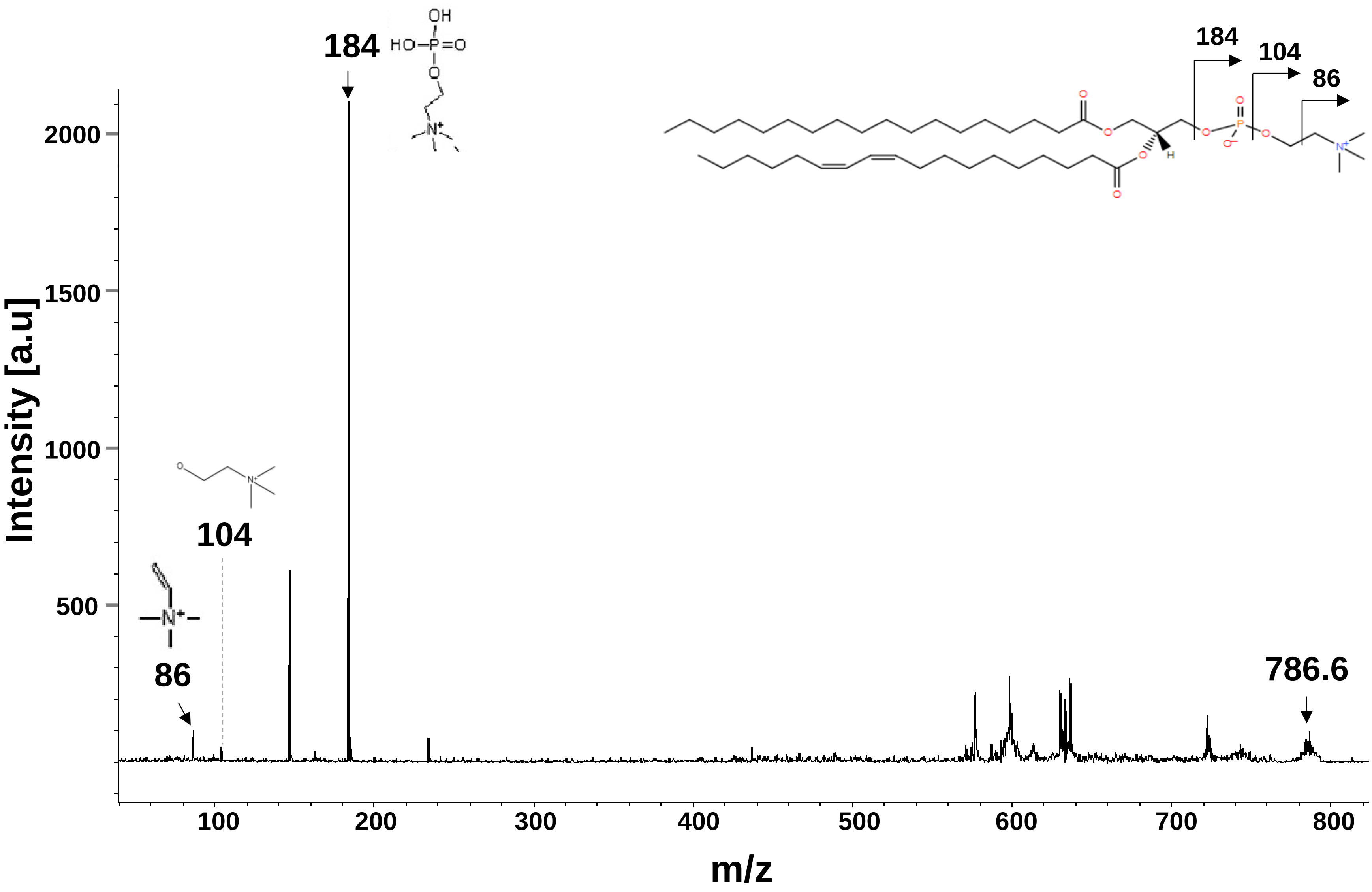
184
104
86
786.6
Intensity [a.u]
m/z
2000
1500
1000
500
100
200
300
400
500
600
700
800

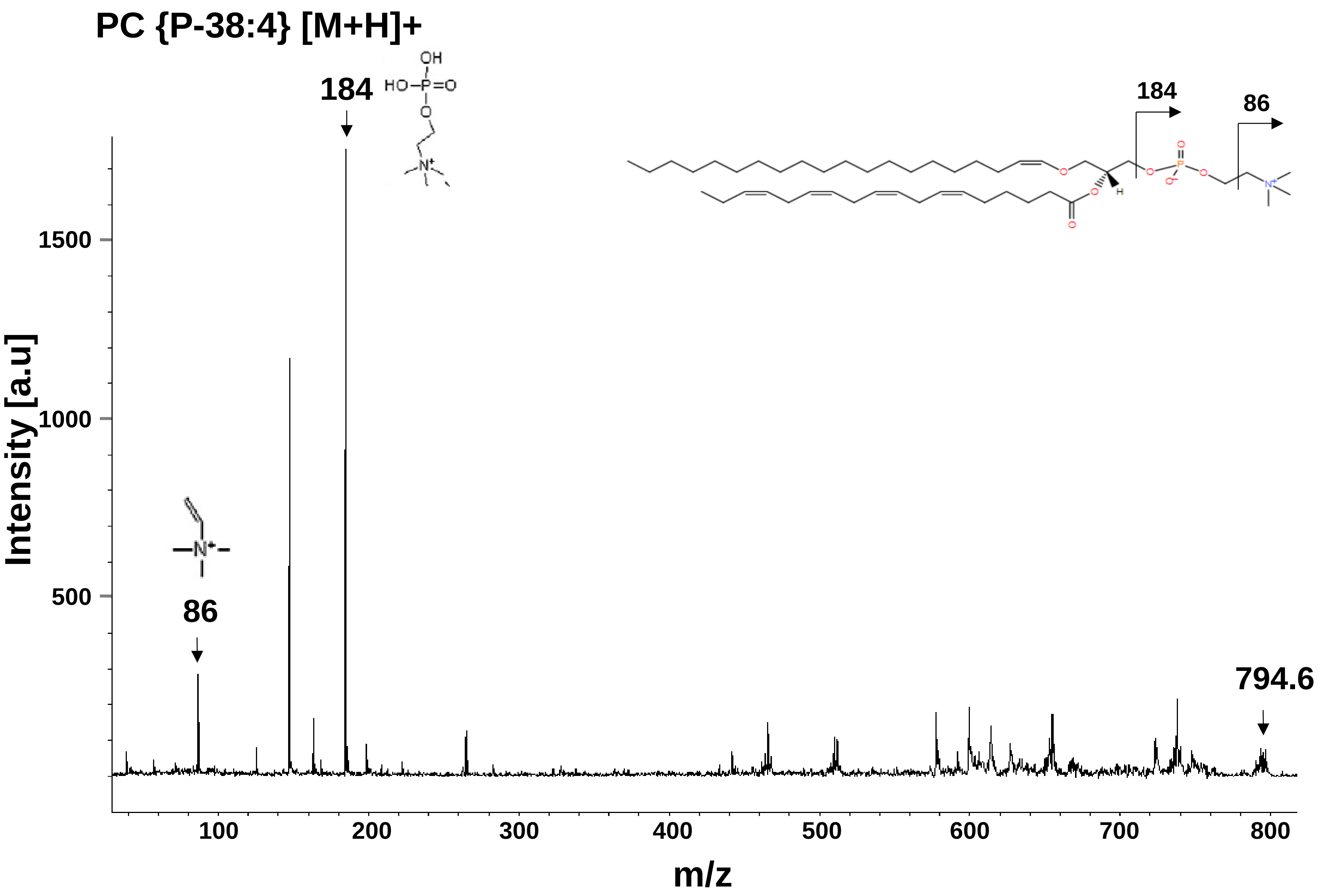
PC {P-38:4} [M+H]+
184
OH
HO–P=O
O
N+
184
86
86
794.6
Intensity [a.u]
1500
1000
500
100
200
300
400
500
600
700
800
m/z

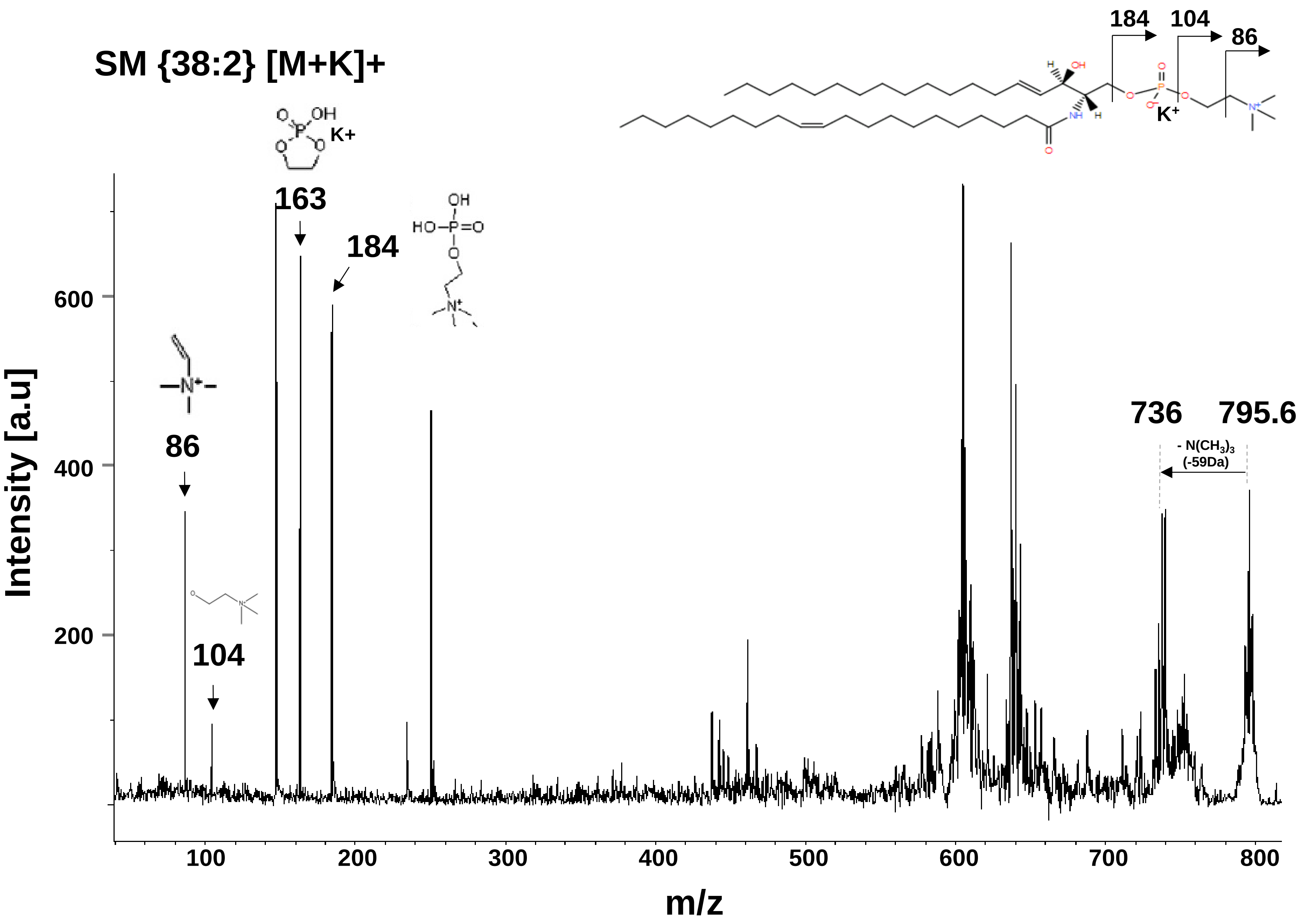

SM {38:2} [M+K]+
184
104
86
K+
163
184
86
104
736
795.6
- N(CH3)3
(-59Da)
600
400
200
Intensity [a.u]
100
200
300
400
500
600
700
800
m/z

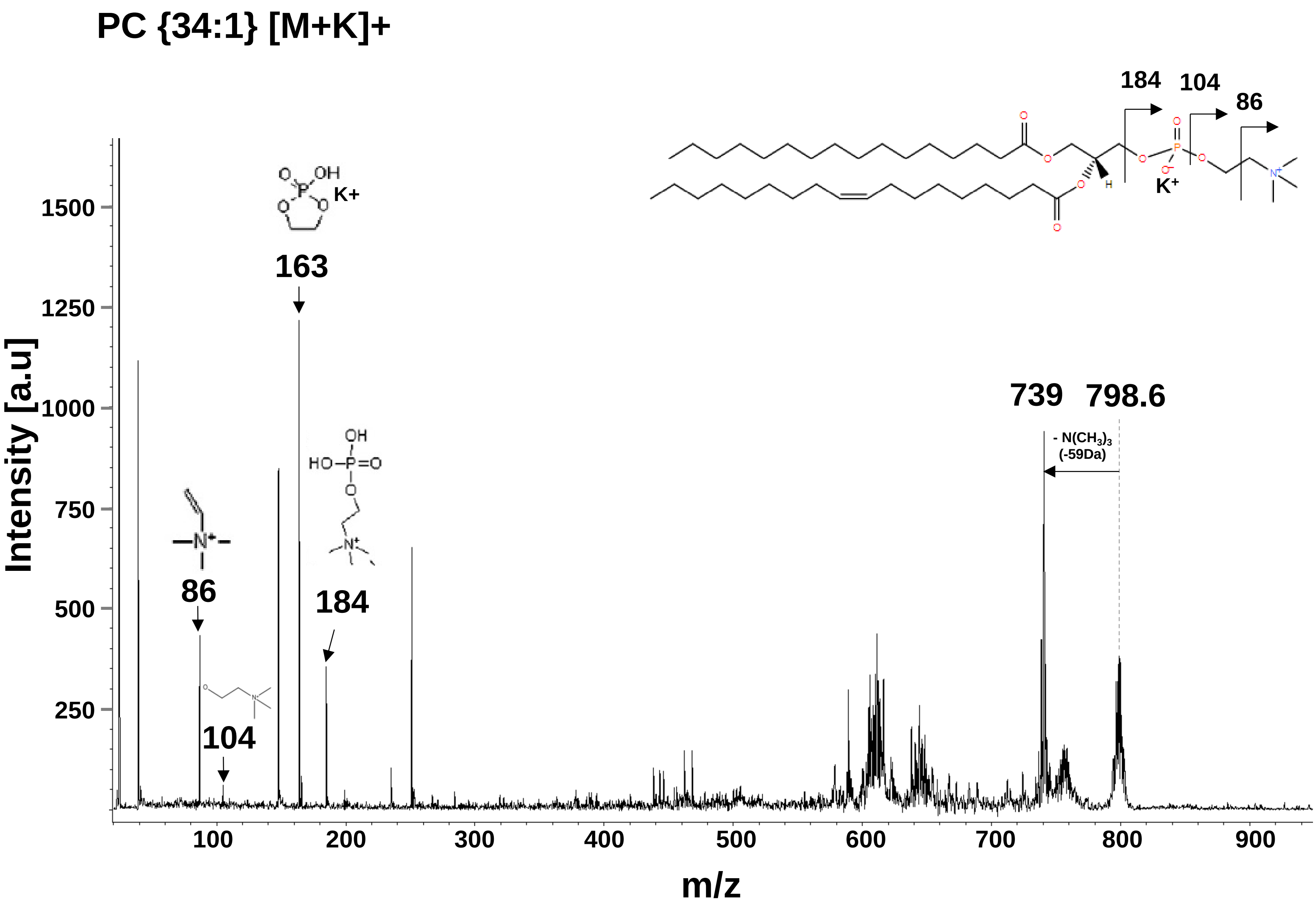
PC {34:1} [M+K]+
184
104
86
K+
K+
163
739
798.6
- N(CH3)3
(-59Da)
86
184
104
Intensity [a.u]
m/z
250
500
750
1000
1250
1500
100
200
300
400
500
600
700
800
900

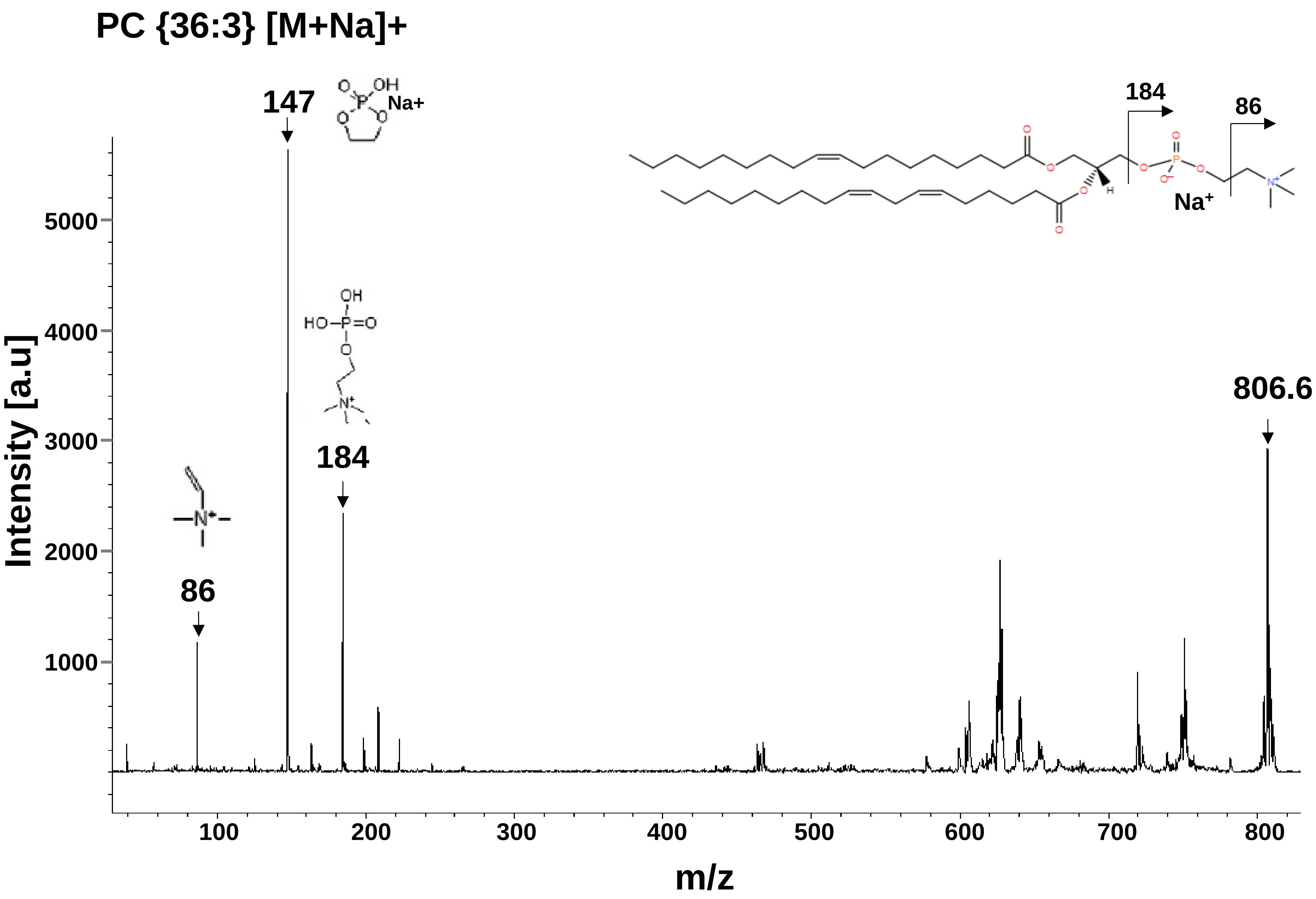

PC {36:3} [M+Na]+
147
Na+
184
86
Na+
806.6
Intensity [a.u]
5000
4000
3000
2000
1000
100
200
300
400
500
600
700
800
m/z

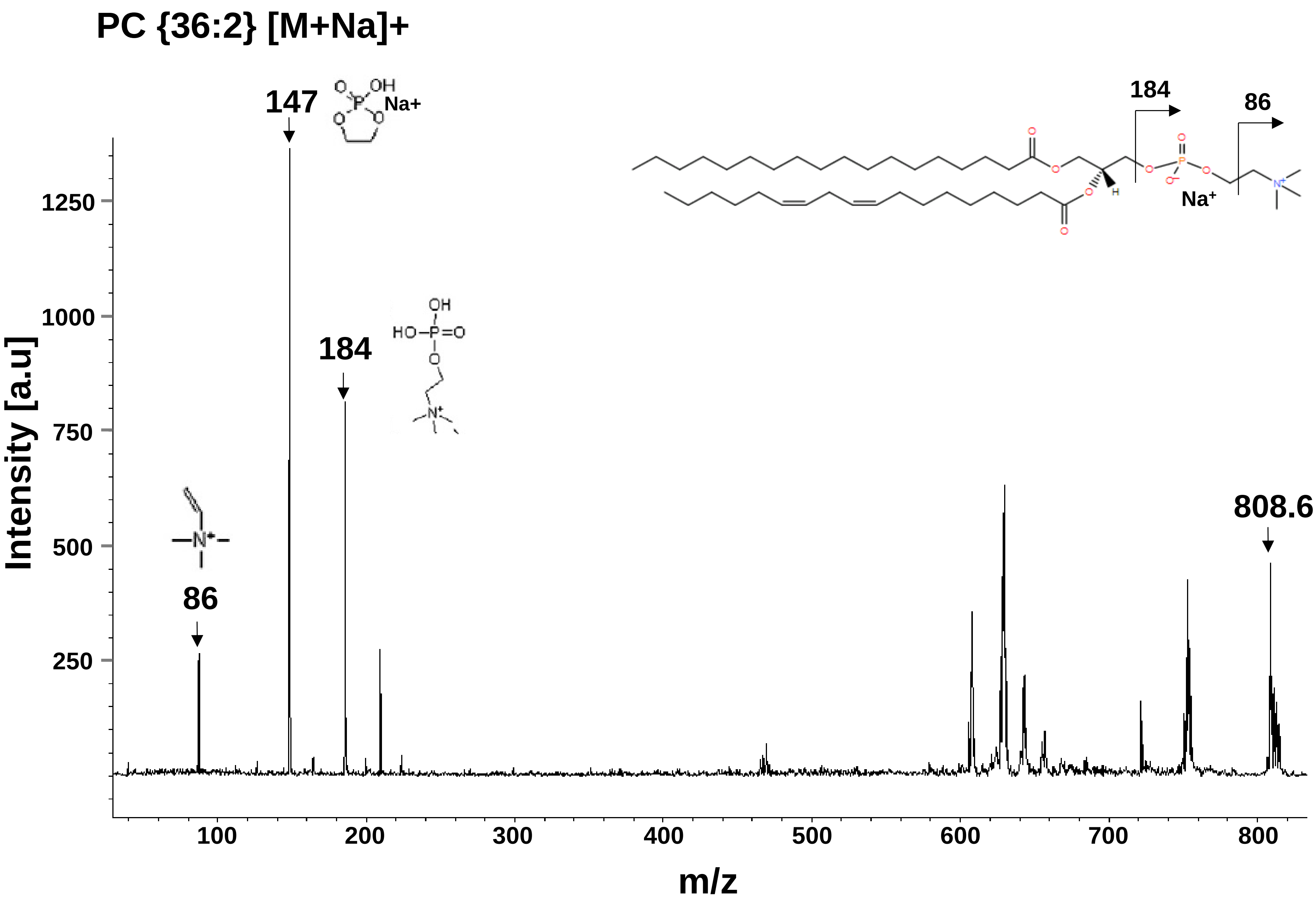

PC {36:2} [M+Na]+
147
Na+
184
86
808.6
Intensity [a.u]
m/z
1250
1000
750
500
250
100
200
300
400
500
600
700
800
Na+

SM {42:1} [M+H]+

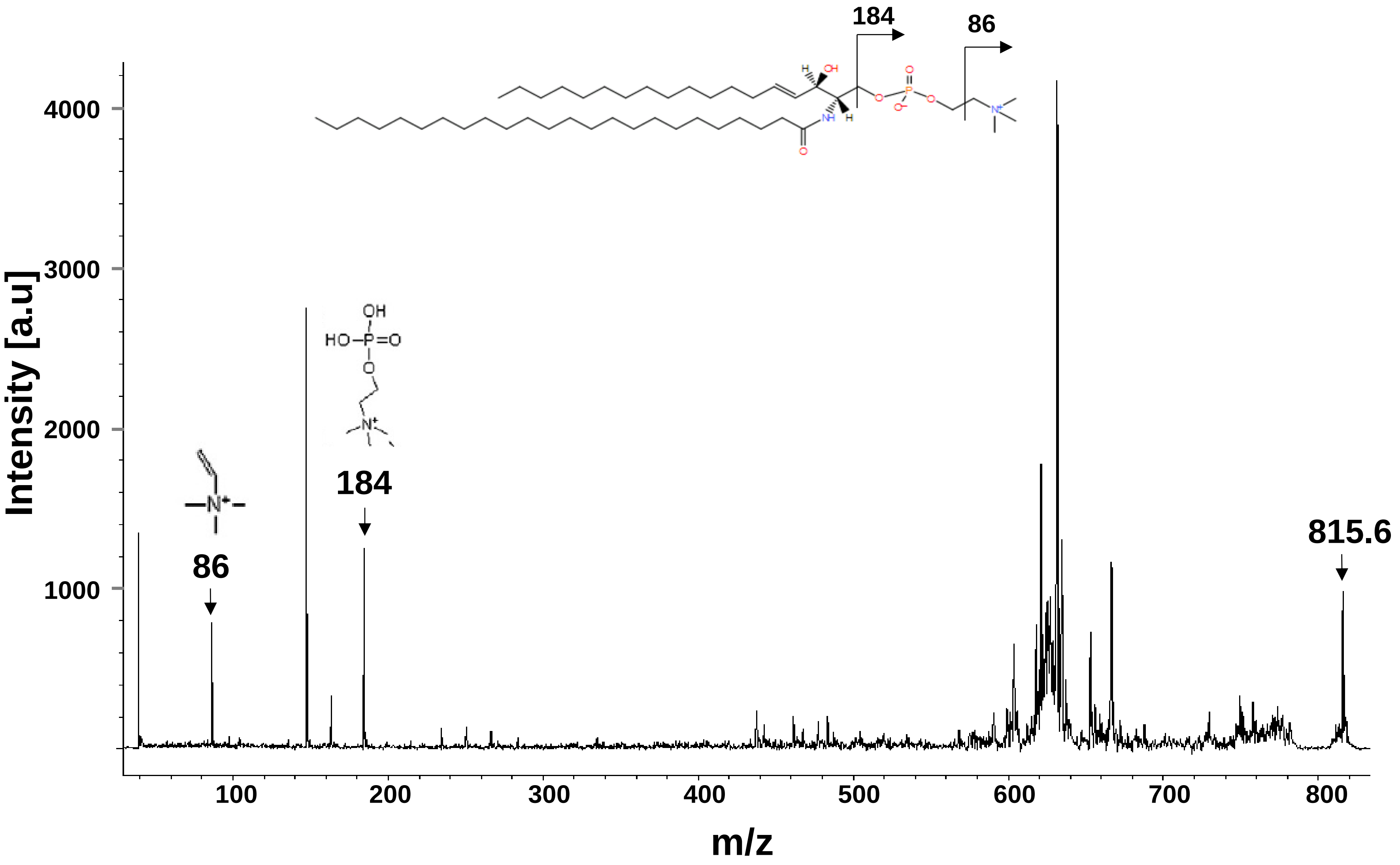
184
86
OH
HO–P=O
O
N+
184
86
815.6
Intensity [a.u]
4000
3000
2000
1000
100
200
300
400
500
600
700
800
m/z

PC {38:7} [M+Na]+

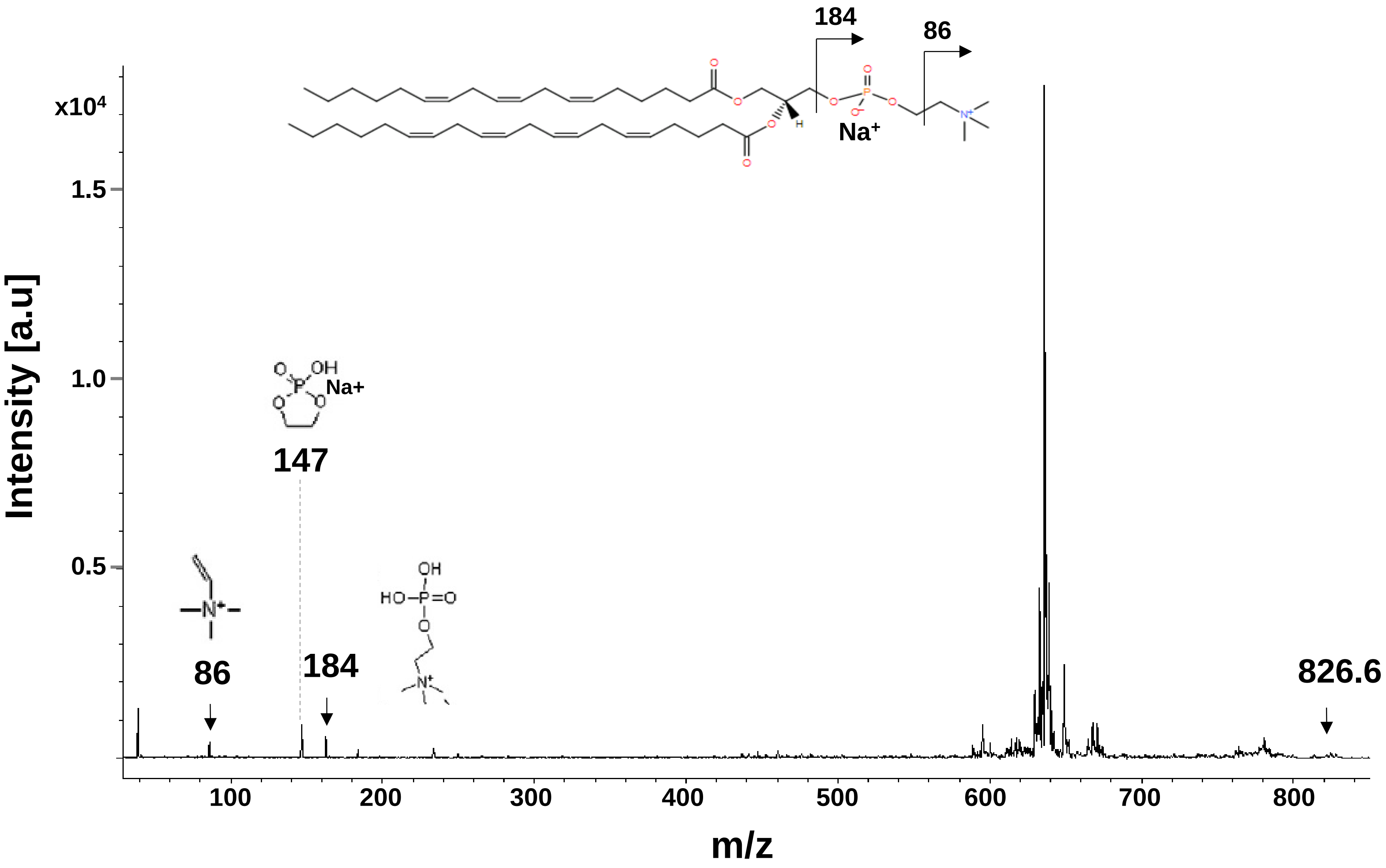

184
86
Na+
x10^4
1.5
1.0
0.5
Intensity [a.u]
Na+
147
86
184
826.6
100
200
300
400
500
600
700
800
m/z

PC {42:6} [M+Na]+

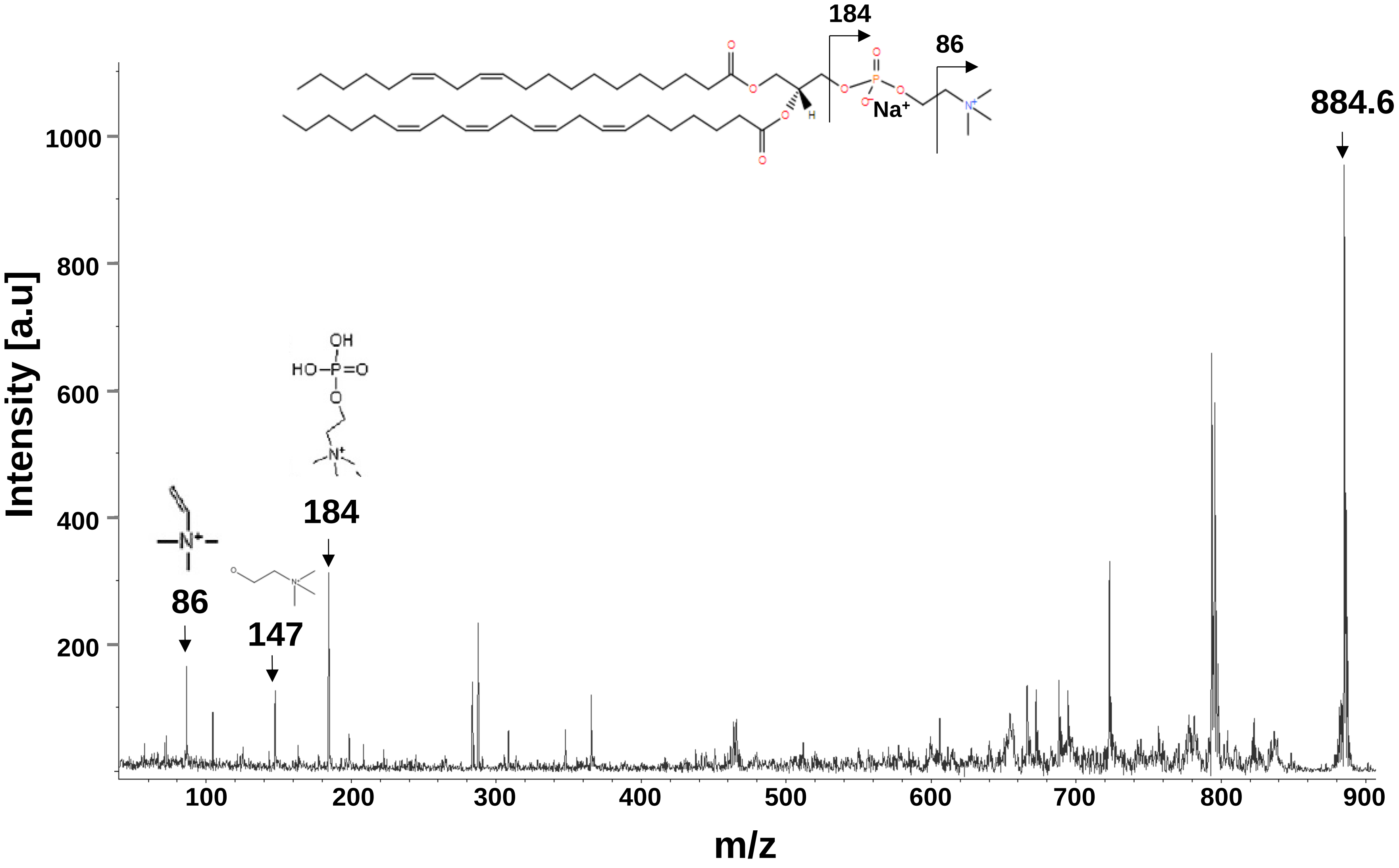
184
86
Na+
884.6
OH
HO–P=O
O
N+
184
86
147
Intensity [a.u]
1000
800
600
400
200
100
200
300
400
500
600
700
800
900
m/z

MS/MS spectrum (negative ion mode)

LPI {18:0} [M-H]-

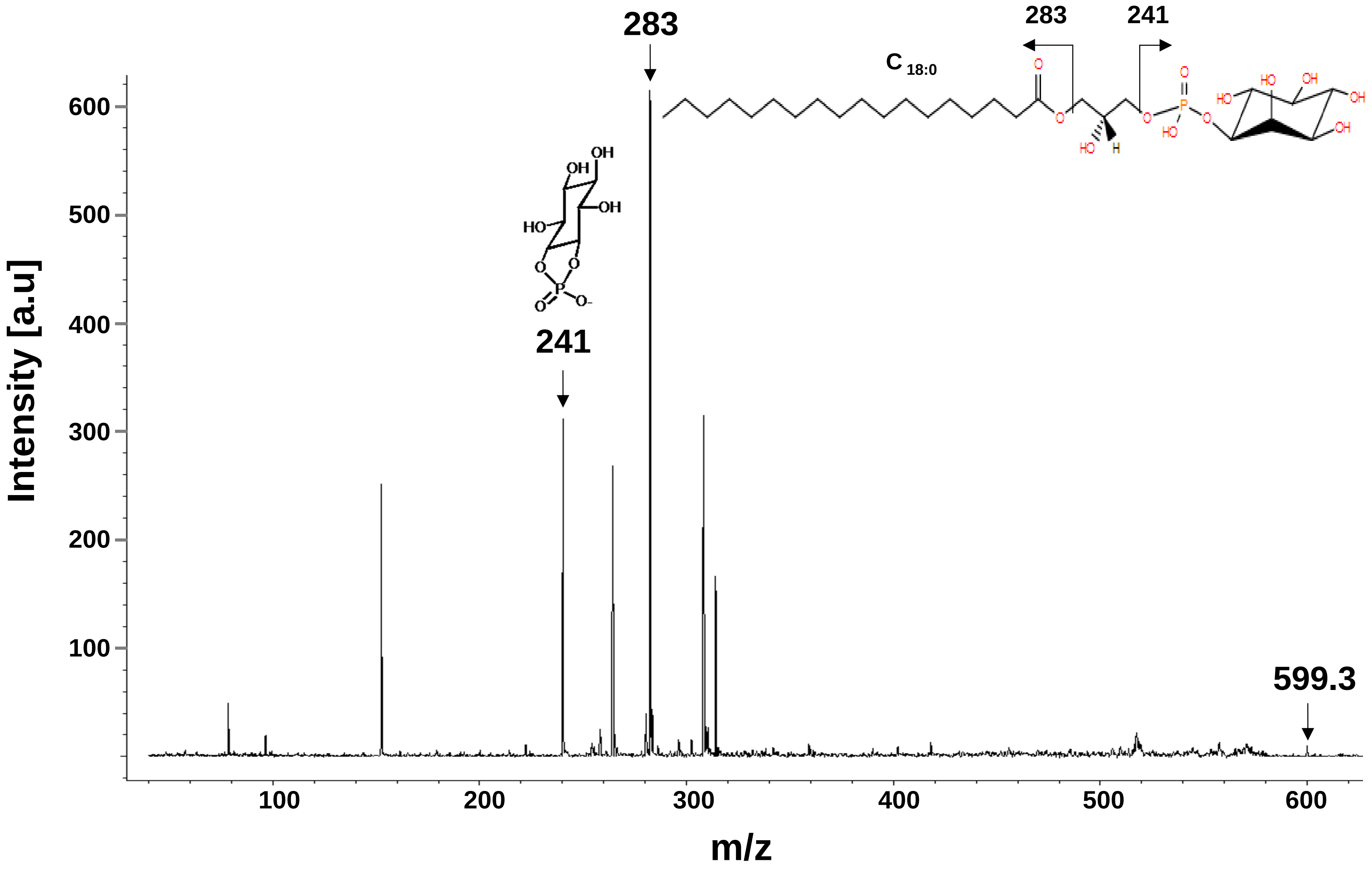
283
241
C 18:0
283
241
599.3
Intensity [a.u]
m/z
100
200
300
400
500
600

PG {P-16:0/14:0} [M-H]-

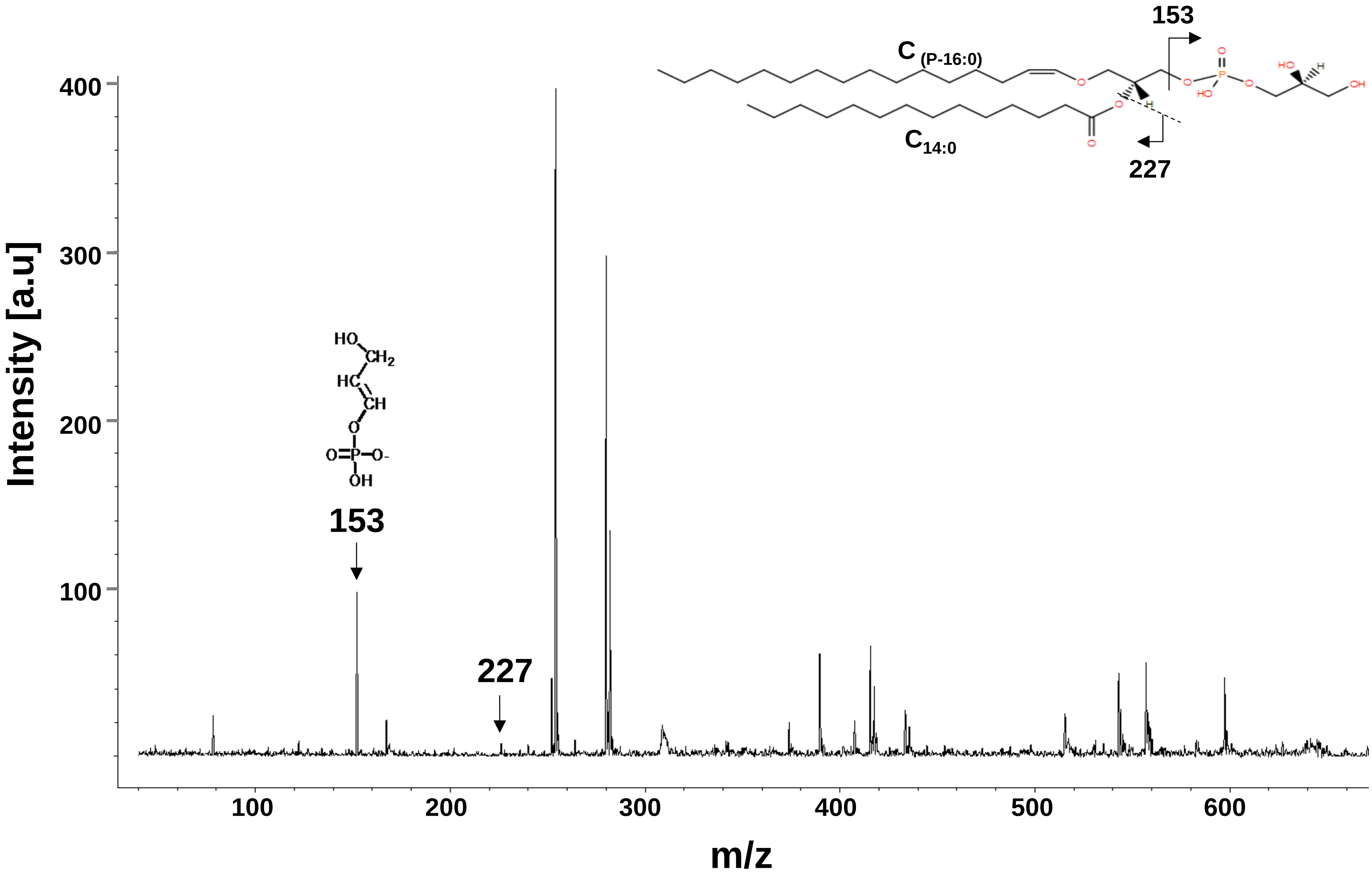

PA {18:2/18:0} [M-H]-

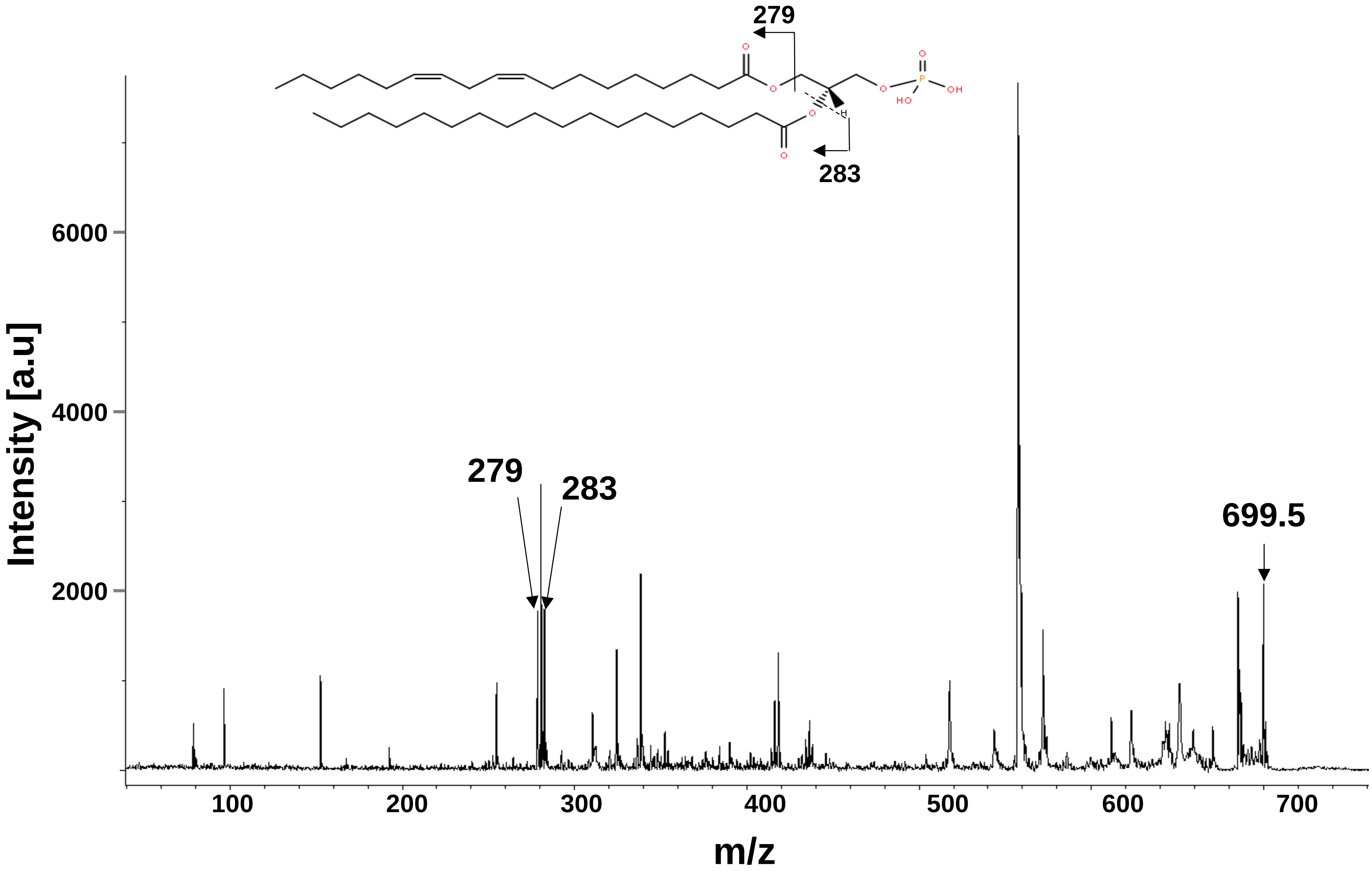

279
283
Intensity [a.u]
6000
4000
2000
279
283
699.5
100
200
300
400
500
600
700
m/z

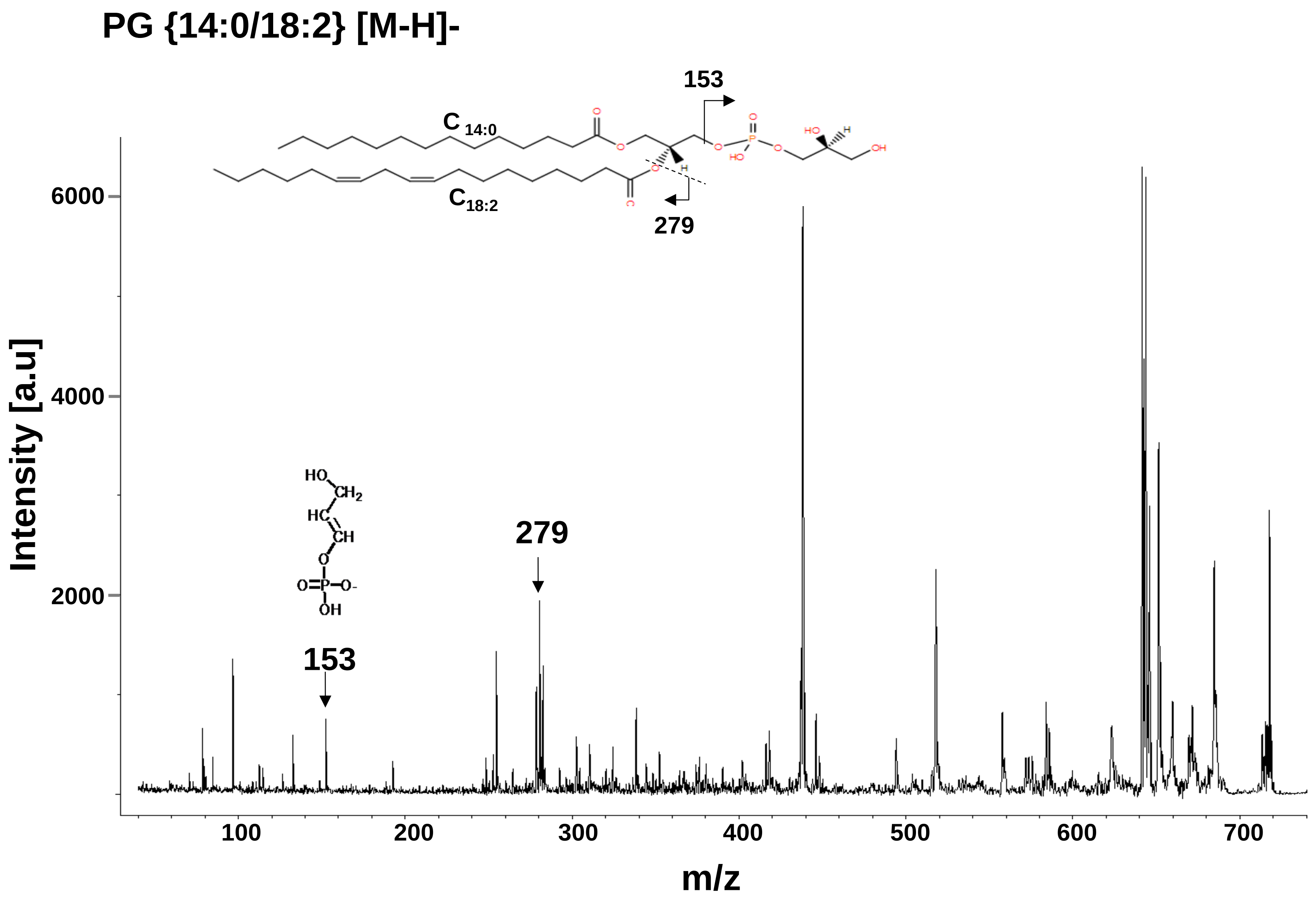
PG {14:0/18:2} [M-H]-
153
C 14:0
C18:2
279
HO
CH2
HC
CH
O
O=P-O-
OH
279
153
6000
4000
2000
Intensity [a.u]
100
200
300
400
500
600
700
m/z

PG {O-16:0/20:4} [M-H]-

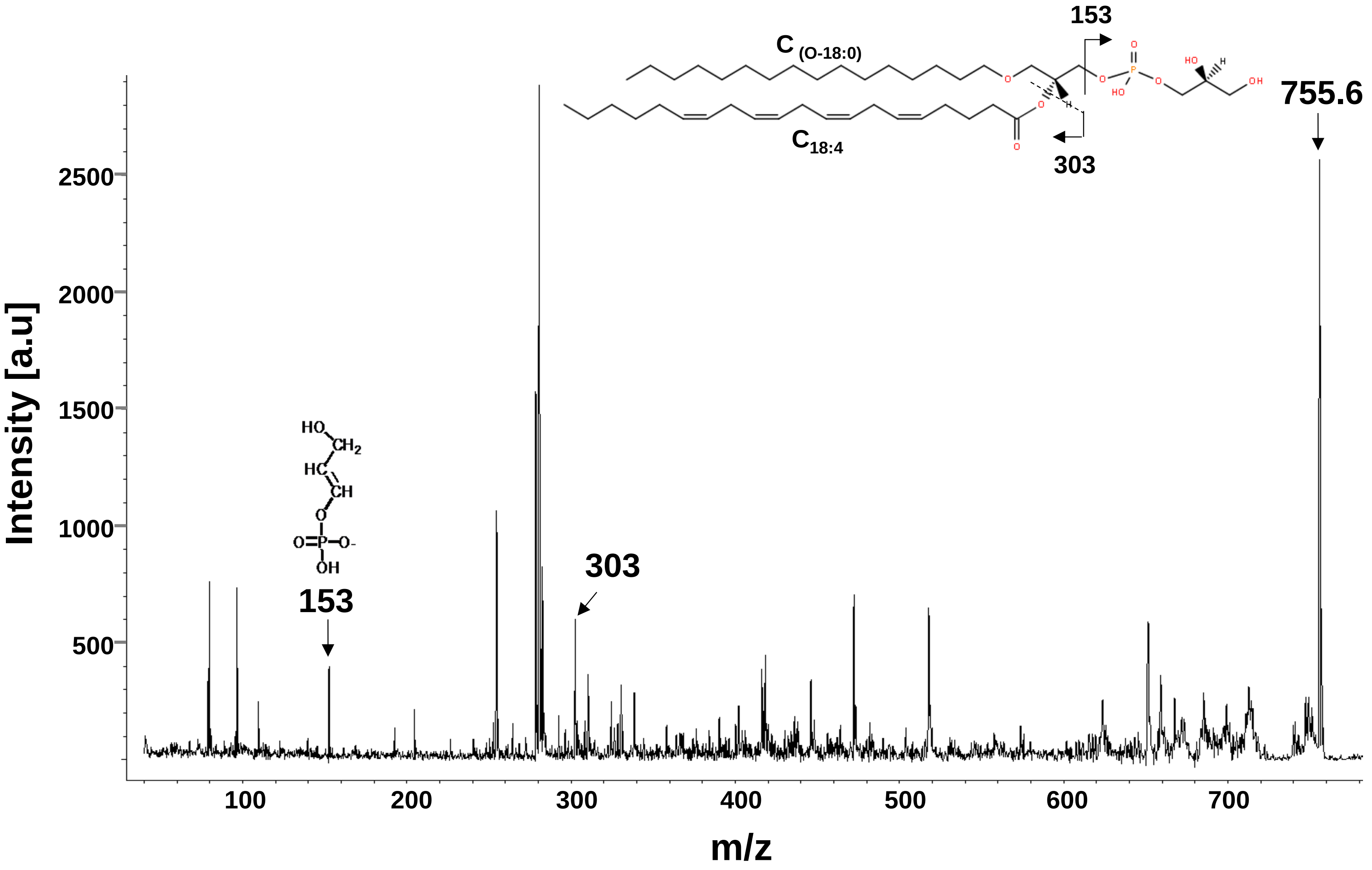

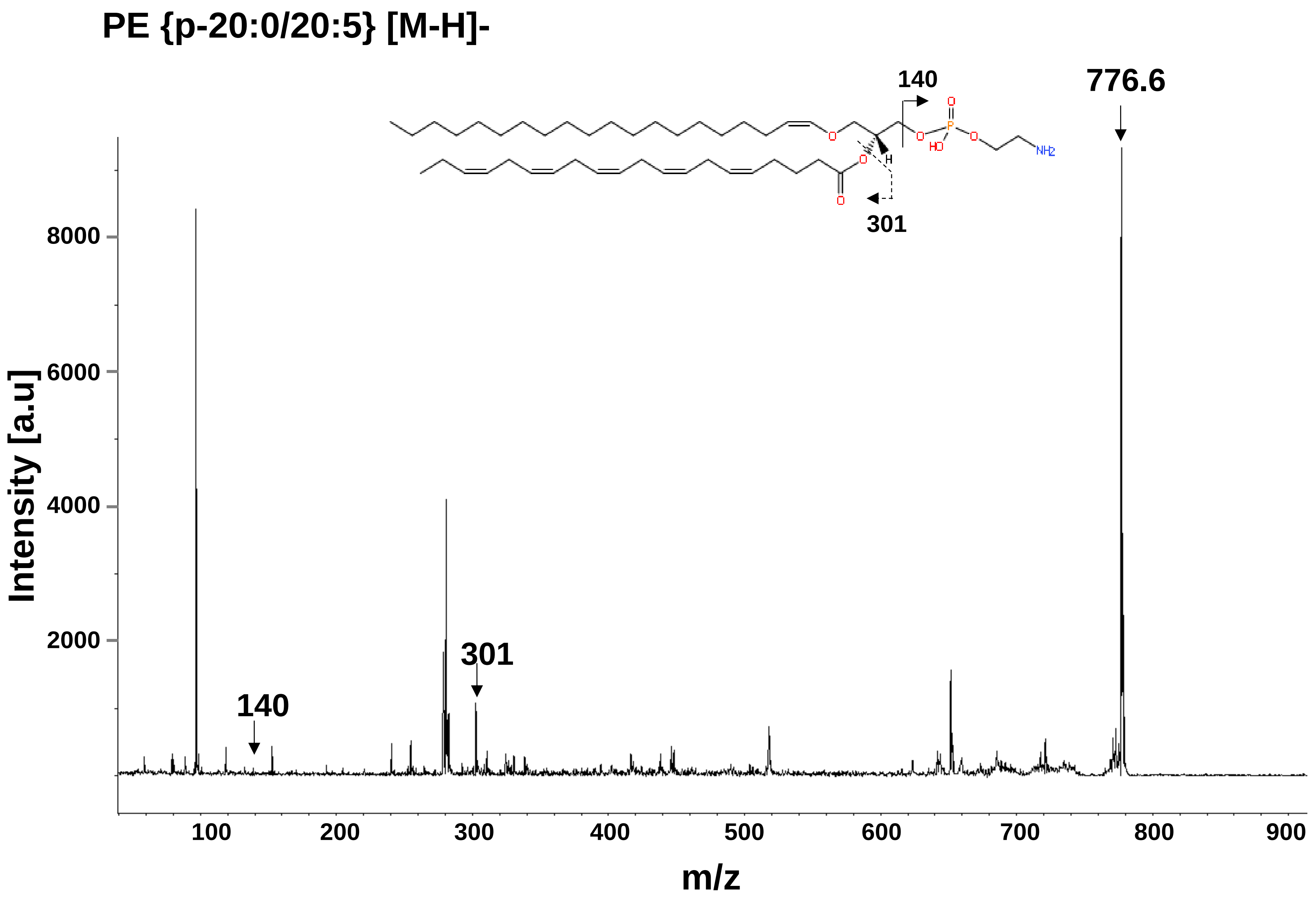
PE {p-20:0/20:5} [M-H]-
776.6
140
301
O
P
HO
NH2
H
Intensity [a.u]
8000
6000
4000
2000
100
200
300
400
500
600
700
800
900
m/z

PI {18:2/16:0} [M-H]-

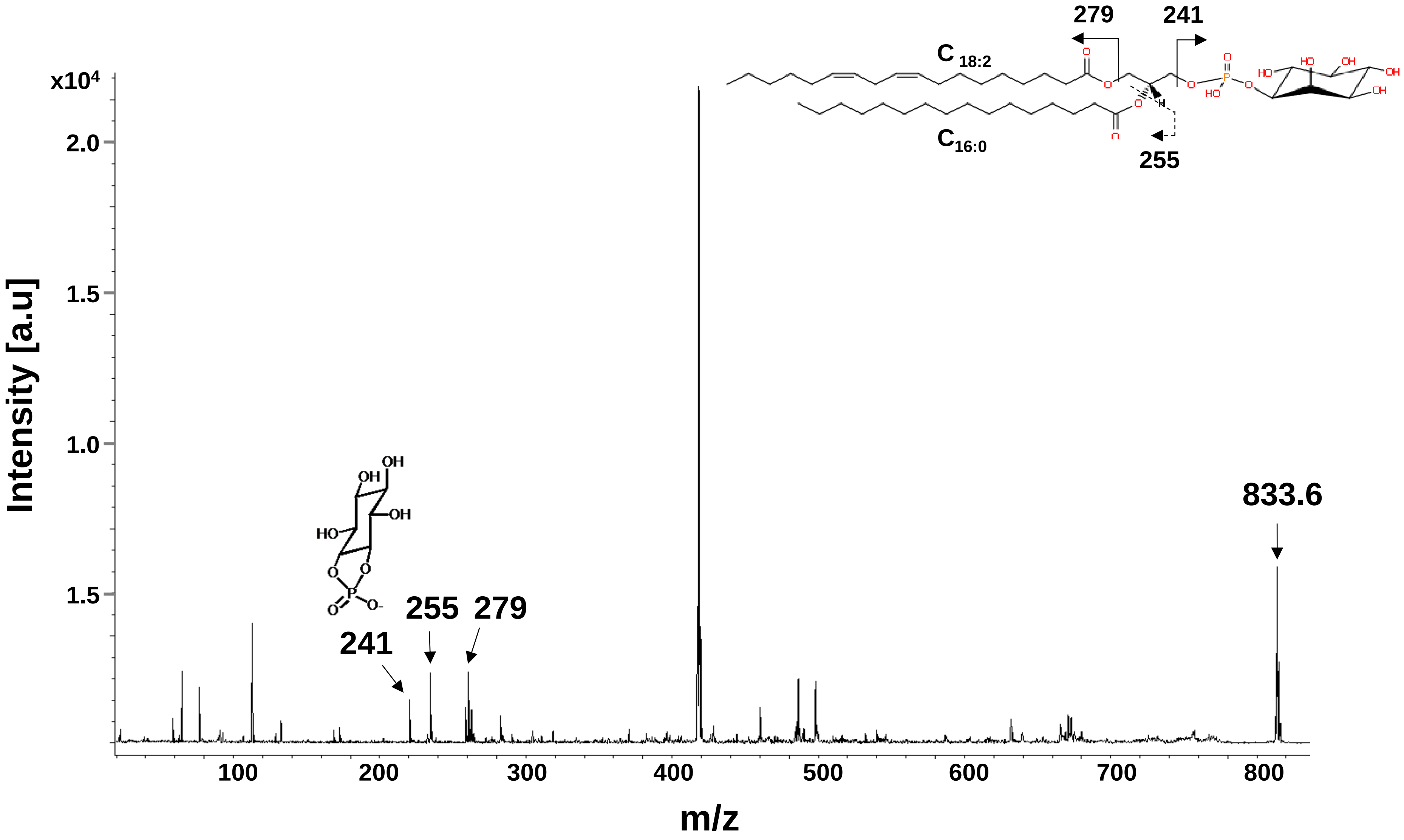
x10^4
2.0
1.5
1.0
1.5
Intensity [a.u]
m/z
100
200
300
400
500
600
700
800
279
241
C 18:2
C16:0
255
833.6
241
255
279

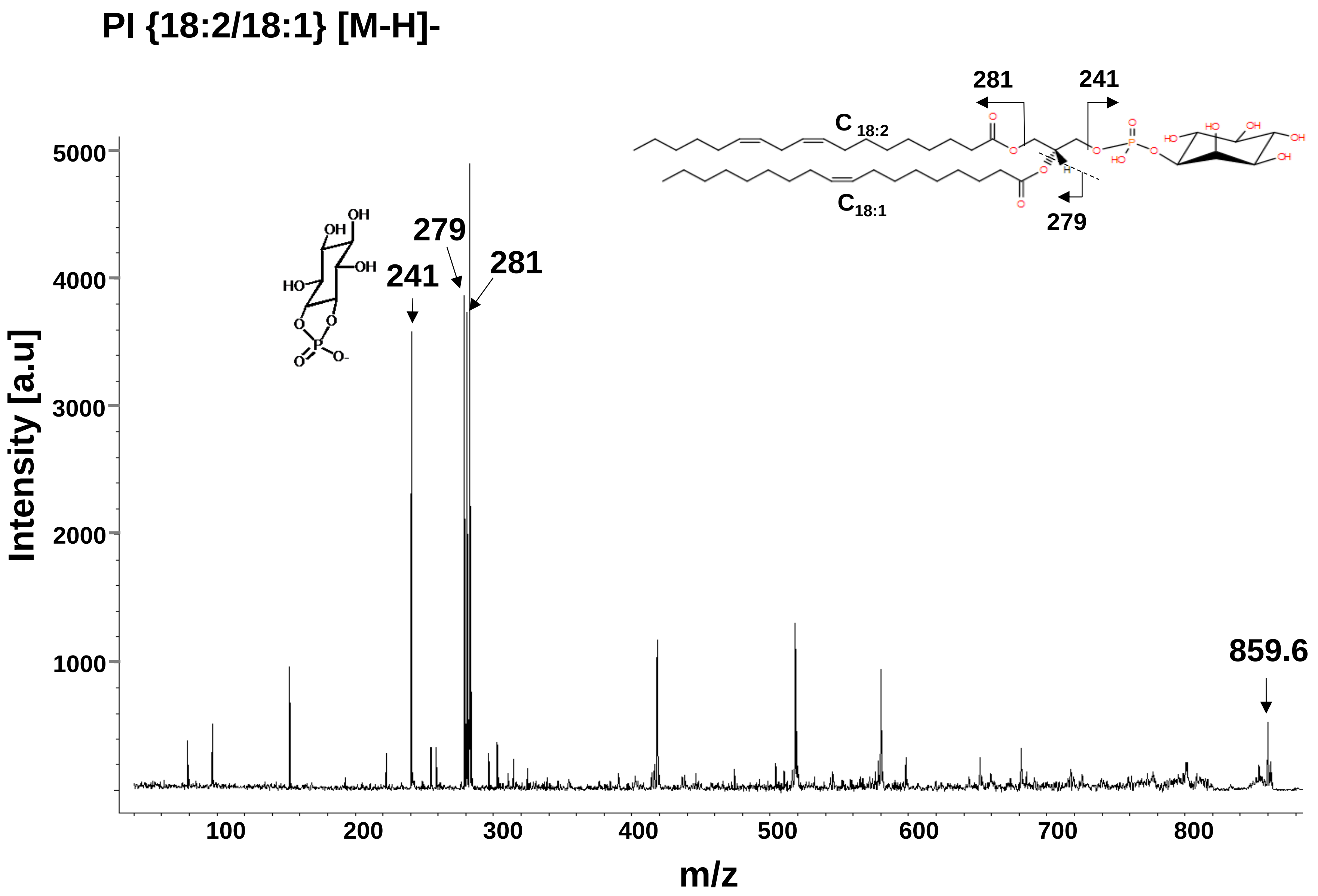
PI {18:2/18:1} [M-H]-
281
241
C 18:2
C18:1
279
OH
OH
OH
HO
O
O
P
O
O-
279
241
281
859.6
5000
4000
3000
2000
1000
Intensity [a.u]
100
200
300
400
500
600
700
800
m/z

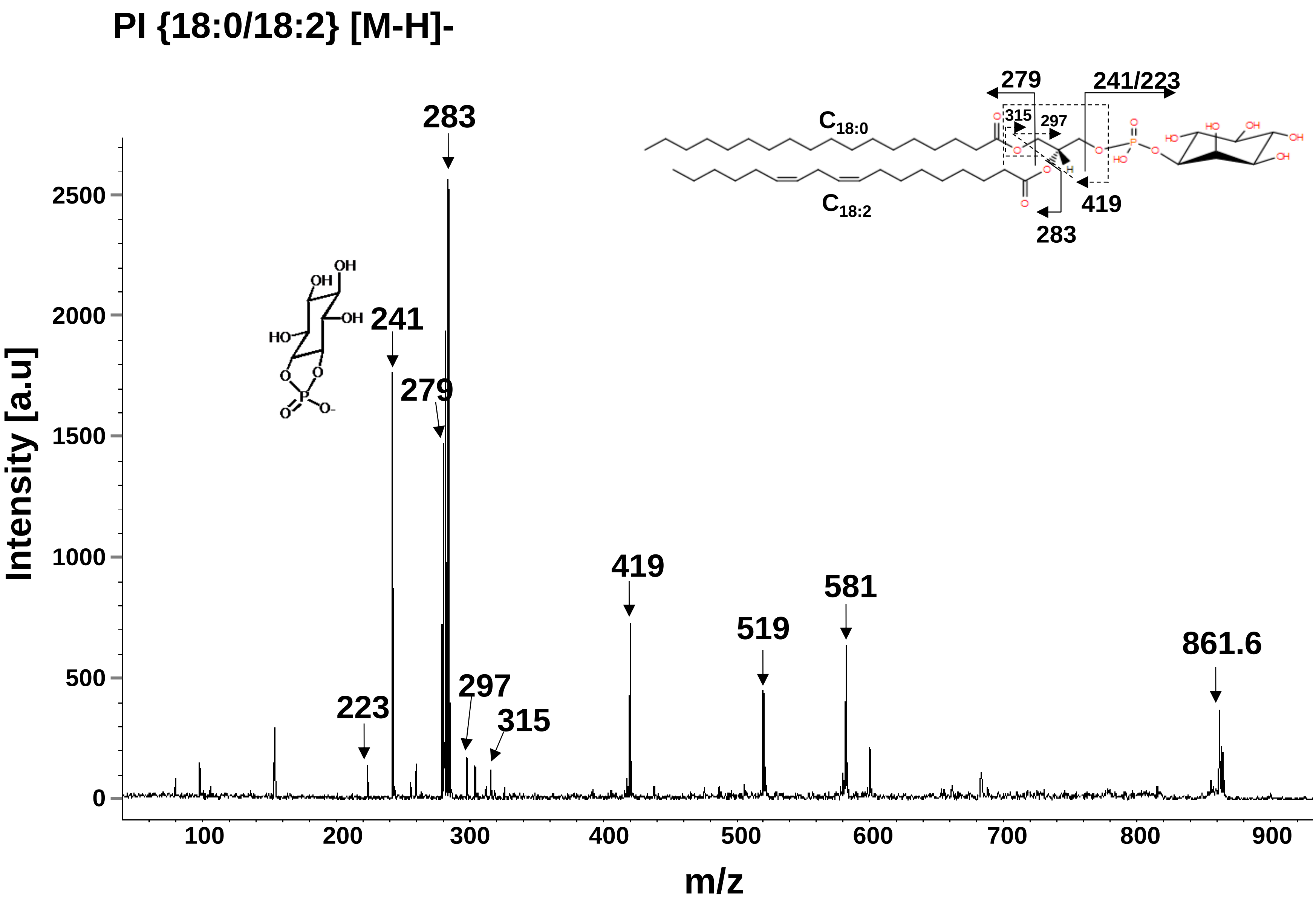
PI {18:0/18:2} [M-H]-
Intensity [a.u]
m/z
283
241
279
223
297
315
419
519
581
861.6
C18:0
C18:2
241/223
0
500
1000
1500
2000
2500
100
200
300
400
500
600
700
800
900

PI {20:4/18:1} [M-H]-

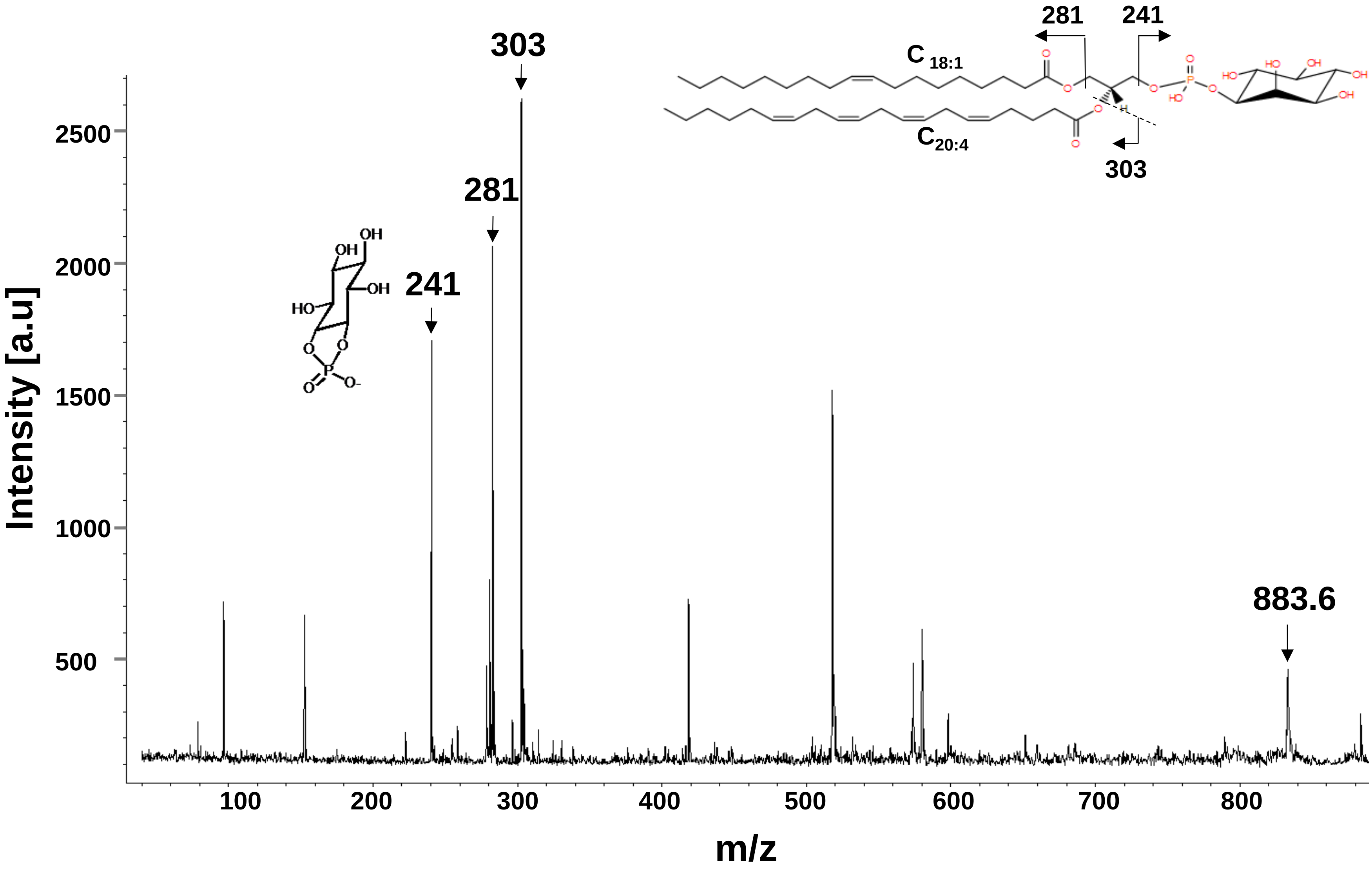
303
281
241
883.6
281
241
C 18:1
C20:4
303
OH
OH
OH
HO
O
O
P
O
O-
2500
2000
1500
1000
500
100
200
300
400
500
600
700
800
Intensity [a.u]
m/z

PI {20:4/18:0} [M-H]-

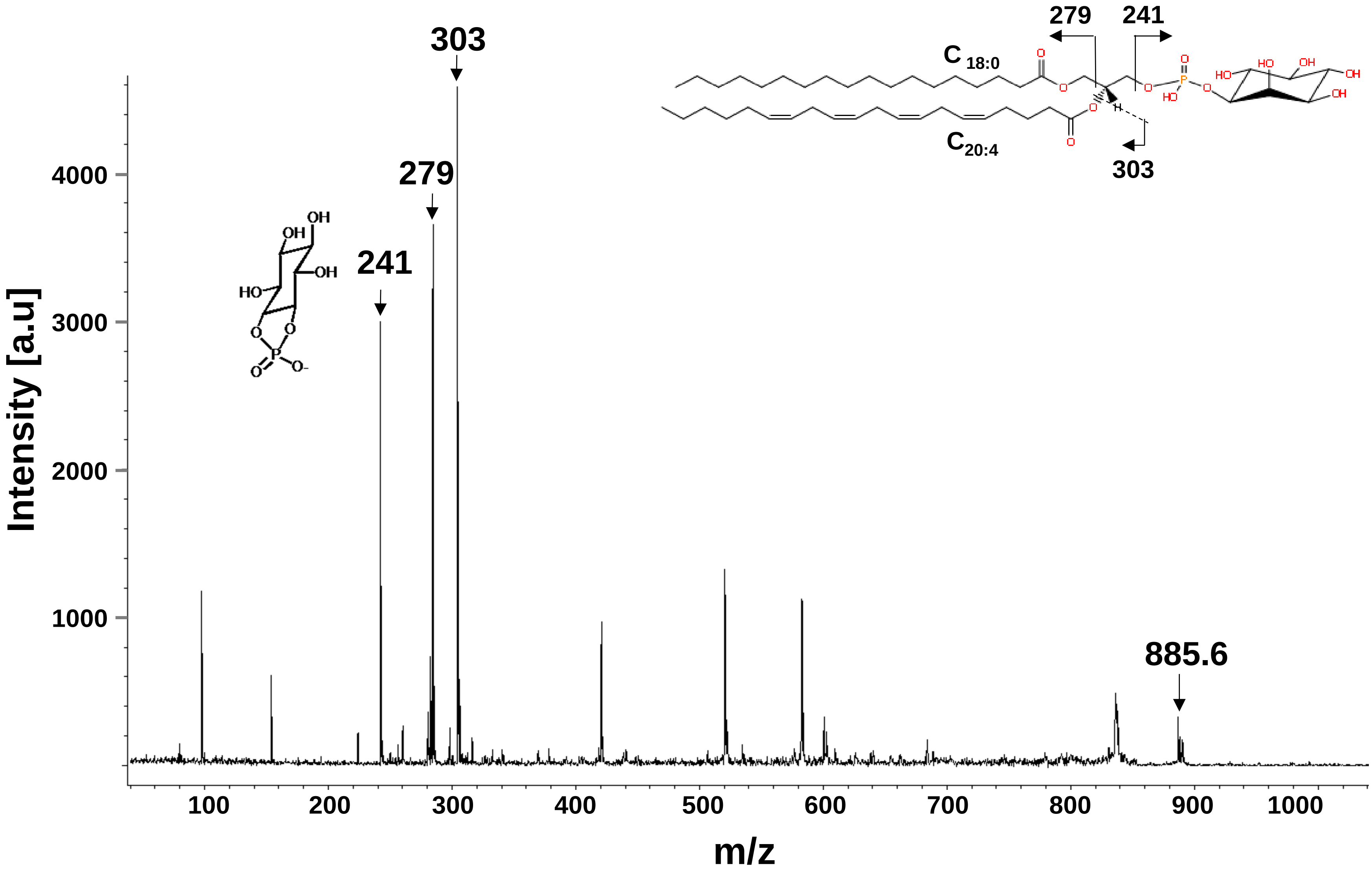

303
279
241
885.6
C 18:0
C20:4
Intensity [a.u]
m/z
4000
3000
2000
1000
100
200
300
400
500
600
700
800
900
1000

PI {20:3/18:0} [M-H]-

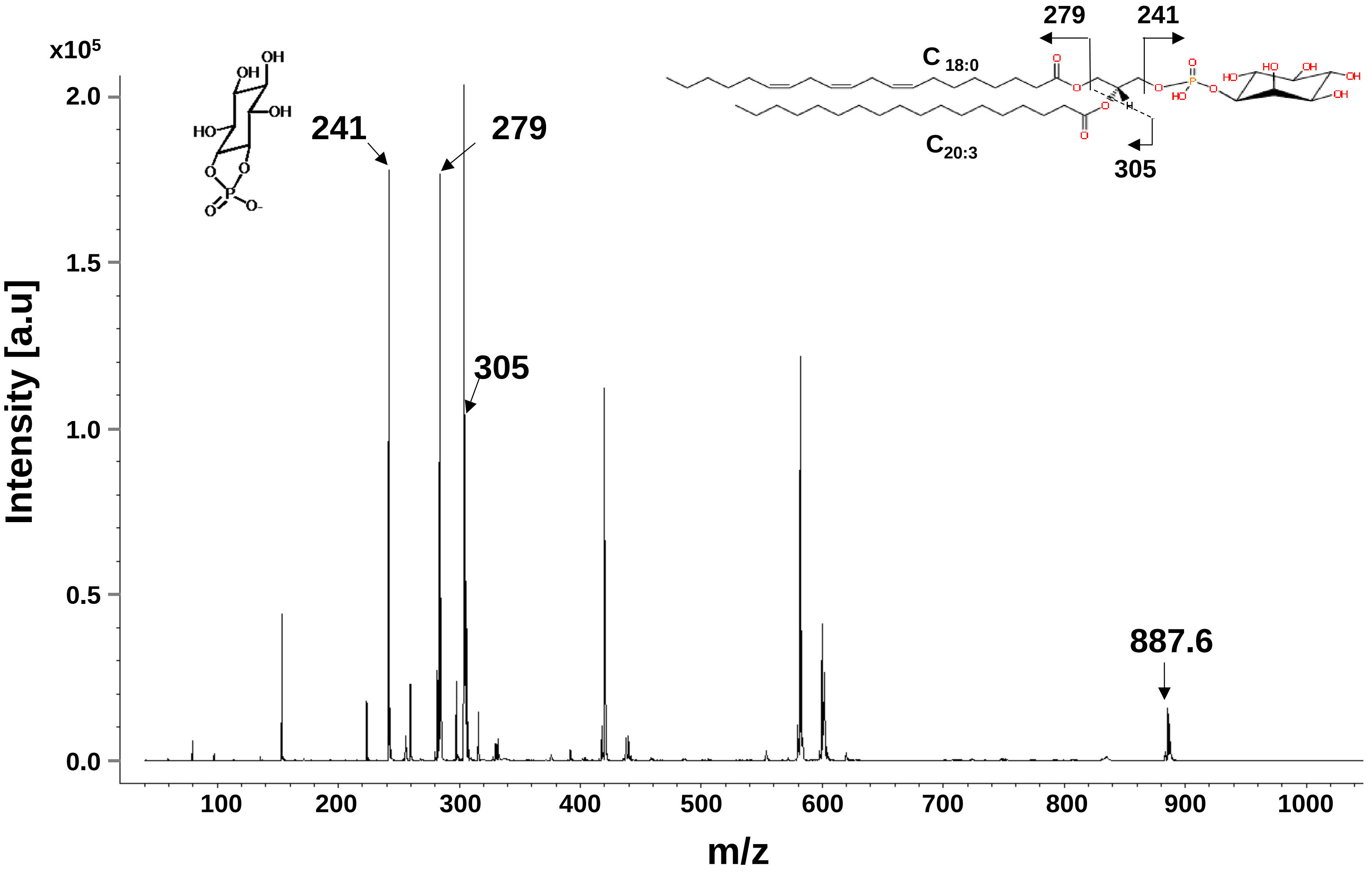

x10^5
Intensity [a.u]
2.0
1.5
1.0
0.5
0.0
100
200
300
400
500
600
700
800
900
1000
m/z
241
279
305
887.6
279
241
C 18:0
C20:3
305
